# Giant optical nonlinearities from Rydberg excitons in semiconductor microcavities

**DOI:** 10.1038/s41467-018-03742-7

**Published:** 2018-04-03

**Authors:** Valentin Walther, Robert Johne, Thomas Pohl

**Affiliations:** 1 Department of Physics and Astronomy, Aarhus University, Ny Munkegade 120, DK 8000 Aarhus, Denmark; 2 Max Planck Institute for the Physics of Complex Systems, Nöthnitzer Str. 38, 01187 Dresden, Germany

## Abstract

The realization of exciton polaritons—hybrid excitations of semiconductor quantum well excitons and cavity photons—has been of great technological and scientific significance. In particular, the short-range collisional interaction between excitons has enabled explorations into a wealth of nonequilibrium and hydrodynamical effects that arise in weakly nonlinear polariton condensates. Yet, the ability to enhance optical nonlinearities would enable quantum photonics applications and open up a new realm of photonic many-body physics in a scalable and engineerable solid-state environment. Here we outline a route to such capabilities in cavity-coupled semiconductors by exploiting the giant interactions between excitons in Rydberg states. We demonstrate that optical nonlinearities in such systems can be vastly enhanced by several orders of magnitude and induce nonlinear processes at the level of single photons.

## Abstract (web summary)

Strong optical nonlinearities in polariton systems open up experiments on quantum-correlated states of light. Here, Walther et al. study Rydberg excitons inside a cavity and show how enhanced nonlinearities could be achieved in such systems.

## Introduction

The achievement of strong coupling between quantum-well excitons and optical photons in semiconductor microcavities^1^ has ushered in new lines of research on exciton-polariton systems. Their unique properties in combination with advanced semiconductor technology^2^ are exploited for the development of novel devices such as next-generation lasers^3^ but also offer a unique platform for fundamental studies of many-body phenomena^4^. Their excitonic component endows such polaritons with interactions that can drive a variety of collective phenomena from condensation^5^ and superfluidity^6^ to solitons^7^ and parametric amplification^8^. Yet, strongly correlated states and nonlinear processes at the level of individual photons^9^ are inherently difficult to realize due to the weak and short-range nature of typical exciton–exciton interactions^4,10^.

Here we describe how one can reach this quantum regime by dressing the photon field of semiconductor microcavities with strongly interacting Rydberg states of excitons, i.e., with semiconductor excitons in excited internal states. Excited states of excitons have been observed in transition metal dichalcogenide (TMDC) monolayers^11^ and in cuprous oxide, where Rydberg states with principal quantum numbers of up to *n* = 25 could be demonstrated^12^. Both materials host excitons with a very large exciton binding energy, which allows to drive transitions within the exciton manifold. In particular, three-level driving schemes offer the possibility to establish conditions of electromagnetically induced transparency (EIT)^13^, enabling strong light matter coupling at suppressed photon losses. Rydberg states feature a number of remarkable properties that are explored and exploited in atomic systems for cavity-QED experiments^14^, quantum simulations and information processing^15^ as well as quantum nonlinear optics^16,17^. Rydberg exciton polaritons therefore offer a promising combination of such new capabilities afforded by the strong interactions between Rydberg states with the technological advantages of semiconductor photonics. Our theoretical framework permits to deduce the associated nonlinear optical response from the rather complex potential surfaces of interacting Rydberg state manifolds, and it indeed yields nonlinearities that exceed those of ground state systems^10^ by many orders of magnitude. Remarkably, this vast enhancement can persist even in the presence of considerable Rydberg state decoherence, up to 10^4^ times stronger then for corresponding atomic Rydberg states^15^. This surprising behavior is traced back to an exciton blockade mechanism not previously discussed for atomic systems.

## Results

### EIT with semiconductor Rydberg states

**Fig. 1:**
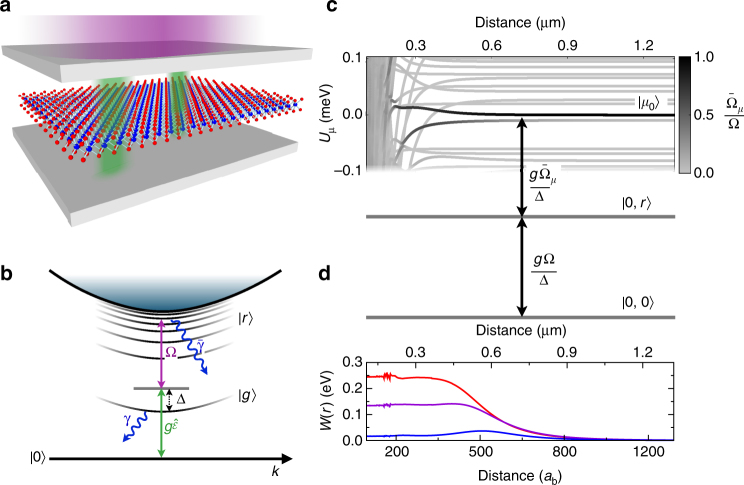
Rydberg excitons in optical microcavities. **a** A Fabry-Pérot cavity (gray) yields strong near resonant coupling of the cavity field $\hat {\cal E}$ (green) to a low-lying exciton state $\left| g \right\rangle$, here it is illustrated for a two-dimensional semiconductor (TMDC monolayer). Another radiation field (purple) provides coupling to excitonic Rydberg states $\left| r \right\rangle$ whose strong interactions generate a nonlinear response to the cavity field. **b** The cavity field generates deeply bound excitons with a coupling strength *g* and single-photon detuning Δ. The additional field provides for two-photon resonant Rydberg state excitation with a Rabi frequency Ω. **c** The resulting two-photon Rabi coupling $\frac{{g{{\rm \Omega }}}}{{{\rm \Delta }}}$ from singly ($\left| {0,r} \right\rangle$) to doubly ($\left| \mu \right\rangle$) excited Rydberg states is strongly influenced by their interactions which cause significant potential energy shifts, *U*_*μ*_(*r*), and distance-dependent coupling strengths ${\bar{\mathrm \Omega }}_\mu (r)$, depicted around the $\left| {\mu _0} \right\rangle = \left| {10p,10p} \right\rangle$ (at *K*^+^) pair state. The gray coloring indicates the relative coupling from stongly- (black) to non-coupling curves (light gray). The resulting photon–photon interaction potential *W*(*r*) is shown in **d** for *g*/2*π* = 5561 GHz^31^, Δ/2*π* = 700 GHz, ground state broadening *γ*/2*π* = 300 GHz, Rydberg state broadening $\bar \gamma /2\pi = 0.3$ GHz and single-photon Rabi coupling Ω/(2*π*) = 10 GHz (blue), Ω/(2*π*) = 20 GHz (purple), and Ω/(2*π*) = 30 GHz (red). The Bohr radius on the lower axis is *a*_*b*_ = 1.11 nm^11^

Figure 1a,b illustrate the considered setup based on near-resonant generation of semiconductor excitons by the photon field, $\hat {\cal E}$, inside an optical microcavity. The quantum field $\hat {\cal E}^\dagger ({\bf{r}})$ creates cavity photons and is defined such that $\hat {\cal E}^\dagger ({\bf{r}})\hat {\cal E}({\bf{r}})$ corresponds to the two-dimensional photon density operator in the plane of the cavity. The cavity photons produce ground state excitons at a coupling strength *g*, which are coupled to an excitonic Rydberg state by another strong externally applied coherent laser field with a Rabi frequency Ω. Importantly, the resulting three-level EIT driving scheme provides strong coupling of light to the excitons, while keeping photon loss at a minimum. Its realization in semiconductors requires quasi-particle band gaps in the optical domain as well as large exciton binding energies. With respect to these requirements, cuprous oxide and TMDC monolayers have emerged as suitable platforms. The actual states that form the EIT scheme shown in Fig. 1b are dictated by the respective band symmetries of the material. In Cu_2_O, the valence and the conduction band have the same parity, such that lowest dipole-allowed transition leads to the $\left| g \right\rangle = \left| {2p} \right\rangle$ exciton of the yellow series at 580 nm^12^. Subsequently, the excitons can be excited to an $\left| r \right\rangle = \left| {ns} \right\rangle$ state, e.g., the $\left| {20s} \right\rangle$ state at a transition wavelength of 50 μm. Semiconducting TMDC monolayers feature even larger exciton binding energies in the range of 0.3–0.7 eV and a remarkably strong light-matter coupling^18^. The excitonic ground state is optically active, such that the $\left| g \right\rangle = \left| {1s} \right\rangle$ exciton can be generated by the optical cavity field, while the excitation to states $\left| r \right\rangle = \left| {np} \right\rangle$ by the external field completes the ladder scheme of Fig. 1b.

We calculate the non-hydrogenic excitonic Rydberg series of such single-layered TMDC materials^19^, accounting for the screened electron–hole interaction^11,20^ as well as Berry curvature effects arising from the band structure topology^21,22^ (Supplementary Note 1). In the following, we consider exciton states obtained for a binding energy of 0.3 eV, equal electron and hole masses of 0.26 *m*_e_^11^ and a Berry curvature of 0.15 nm^2^^22^. At the *K*^+^- and the *K*^−^-points of the Brillouin zone, the calculated two dimensional excitonic wavefunctions $\left| {\phi _{n,m}} \right\rangle$ are fully characterized by the principal quantum number *n* (radial solution) and the angular component *m* = 0, …, ±(*n* − 1), which determines the optical selection rules Δ*m* = ±1. We refer to the states with *m* = 0 as *s*-states and to $\left| m \right| = 1$ as *p*-states. As one important consequence of screening and Berry curvature, the degeneracy of the excited states with different *m* is lifted^21^—a key requirement for many optical schemes. In Cu_2_O, the degeneracy with respect to angular momentum is lifted by anharmonic contributions to the band dispersion^23^.

### Interactions of semiconductor Rydberg states

The nature of the interaction between excitons in excited internal states can differ significantly from that of short-range collisions between exciton ground states^4^. Due to its enormous strength, the interaction between Rydberg excitons typically becomes already relevant at such large distances where exchange effects^24^ are negligible and direct electrostatic interactions play the dominant role. In fact, the occurrence of Rydberg exciton pairs with smaller distances is prevented by an excitation blockade due to the interactions which shift the energy of the doubly excited state far out of resonance^25^. As shown in Supplementary Note 6, for typical parameters the resulting blockade radius indeed exceeds the so-called LeRoy radius^26^ beyond which exchange effects play no significant role. Consequently, the interaction potential has to be determined non-perturbatively by diagonalizing the dipole–dipole interaction operator that couples different pair product states $\left| {\phi _i,\phi _j} \right\rangle = \hat X_i^\dagger \hat X_j^\dagger \left| 0 \right\rangle$ (Supplementary Note 2), where $\hat X_i$ creates a Rydberg exciton in state $\left| {\phi _i} \right\rangle = \left| {\phi _{n,m}} \right\rangle$ from the semiconductor ground state $\left| 0 \right\rangle$. Note that this treatment discards angular momentum state mixing due to the discrete lattice structure^27^, which for excited Rydberg states becomes negligibly small compared to the state mixing induced by the dipole–dipole interactions. The diagonalization yields a set of interaction potentials as shown in Fig. 1c. The resulting interaction Hamiltonian can be written as

1 $$\hat U = {\int} {\rm d}^2{\bf{r}\,}{\rm d}^2{\bf{r{\prime}}}\mathop {\sum}\limits_\mu {\kern 1pt} U_\mu \left( {\left| {{\bf{r}} - {\bf{r{\prime}}}} \right|} \right)\hat X_\mu ^\dagger \left( {{\bf{r}},{\bf{r{\prime}}}} \right)\hat X_\mu \left( {{\bf{r}},{\bf{r{\prime}}}} \right)$$

in terms of the operators $\hat X_\mu ^\dagger ({\bf{r}},{\bf{r{\prime}}})$ = $\mathop {\sum}\nolimits_{ij} {\kern 1pt} c_{ij,\mu }^ \ast \hat X_i^\dagger ({\bf{r}})\hat X_j^\dagger ({\bf{r{\prime}}})$ that create an exciton pair state $\left| \mu \right\rangle$ corresponding to a given potential curve $U_\mu \left( {\left| {{\bf{r}} - {\bf{r{\prime}}}} \right|} \right)$ for two excitons at positions **r** and **r**′. Note that the elements $c_{ij,\mu }^ \ast$ of the two-exciton eigenstate also play an important role since they determine the effective optical coupling strength ${\bar{\mathrm \Omega }}_\mu (r)$ to a given pair state $\left| \mu \right\rangle$, which is indicated by the gray shading of the potential curves in Fig. 1c. The distance dependence arises from the distance-dependent mixing of Rydberg states with different single-exciton Rabi couplings Ω_*i*_ by the dipole–dipole interaction. Even though there are many potential curves, only a few of them couple to the applied laser field due to the optical dipole selection rules and the interaction-induced mixing of optically active and inactive product states $\left| {\phi _i,\phi _j} \right\rangle$. In fact, it is the combination of the large energy shifts by the interaction potentials *U*_*μ*_(*r*) and the spatially dependent coupling strengths ${\bar{\mathrm \Omega }}_\mu (r)$ which gives rise to the nonlinear optical response discussed below.

### Nonlinear optical response

The emergence of a nonlinear optical response is readily understood within a simplified picture known from atomic systems^16,17^, where one assumes vanishing Rydberg state decay ($\bar \gamma = 0$) and considers only a single pair state $\left| {\mu _0} \right\rangle$ with a van der Waals interaction potential $U_{\mu _0}\sim r^{ - 6}$ and negligible state mixing. By tuning the frequency of the Rydberg excitation laser onto two-photon resonance, one can establish EIT conditions within a frequency window ${{\rm \Omega }}^2{{\rm /}}\left| {{\rm \Gamma }} \right|$ determined by the Rydberg excitation Rabi frequency Ω and Γ = *γ* − *i*2Δ, which is given by the decay rate *γ* and the single-photon detuning Δ from the low-lying exciton line (see Fig. 1b). Therefore, the otherwise high optical susceptibility originating from the strong exciton-cavity coupling can be greatly reduced by resonant Rydberg state coupling. This effect can be traced back to the formation of dark-state polaritons^13^ whose coherence properties in the present case are predominantly limited by the Rydberg state linewidth rather than the large decay rate of the low-lying exciton state. The strong interactions between Rydberg states can, however, drastically modify this picture. As discussed above, the level shift, $U_{\mu _0}(r)$, induced by a single Rydberg excitation can be sufficient to inhibit any further excitation in its vicinity. This interaction-induced Rydberg excitation blockade thus prevents the establishment of EIT conditions and thereby exposes the strong optical response of the low-lying transition. The interplay of interaction-induced level shifts and EIT, therefore, provides a simple mechanism for the emergence of strong, spatially nonlocal optical nonlinearities^28^ that has been demonstrated in a number of recent experiments in atomic systems^16,17^.

In a semiconductor, however, the much larger energy scales for the light-matter coupling and stronger decoherence prompt the necessity of a more advanced theory that accounts for the collective coupling to a large manifold of strongly interacting Rydberg exciton states. To this end, we determine the nonlinearity for coherent light fields, described by the amplitude ${\cal E}({\bf{r}})$, from the polarization

2 $${\cal P}({\bf{r}}) = \chi ^{({\bf{1}})}{\cal E}({\bf{r}}) + {\int} {{\rm d}}{\bf{r{\prime}}}\chi ^{(3)}\left( {\left| {{\bf{r}} - {\bf{r{\prime}}}} \right|} \right)\left| {{\cal E}({\bf{r{\prime}}})} \right|{\cal E}({\bf{r}}),$$

by solving the many-body steady state of the driven interacting excitons to leading order in the Rydberg state densities. As derived in more detail in Supplementary Note 3, the Heisenberg equation for the Rydberg excitons can be written as

3 $$\begin{array}{*{20}{l}} {\partial _t\hat X_k\left( {\bf{r}} \right)} \hfill & = \hfill & {\frac{{g{{\rm \Omega }}_k}}{{{\rm \Gamma }}}{\cal E}({\bf{r}}) - \frac{{{{\rm \Gamma }}_k}}{2}\hat X_k({\bf{r}}) - \frac{{{{\rm \Omega }}_k}}{{2{{\rm \Gamma }}}}\mathop {\sum}\limits_i {\kern 1pt} {{\rm \Omega }}_i\hat X_i({\bf{r}})} \hfill \\ {} \hfill & {} \hfill & { - i\mathop {\sum}\limits_{i \le j,i{\prime}} {\int} {{\rm d}}{\bf{r{\prime}}}\hat X_{i{\prime}}^\dagger ({\bf{r{\prime}}})\frac{{V_{i{\prime}k,ij}^{\rm dd}\left( {\left| {{\bf{r{\prime}}} - {\bf{r}}} \right|} \right)}}{\hbar }\hat X_i({\bf{r{\prime}}})\hat X_j({\bf{r}}).} \hfill \end{array}$$

Here, the decay, dephasing, and decoherence of the excitonic ground and Rydberg state are accounted for by the rate constants *γ* and $\bar \gamma _k$, respectively. Besides the single-photon detuning Δ of the lower transition and Γ = *γ* − 2*i*Δ, we have defined the two-photon detunings Δ_*k*_ for a given excited state with ${{\rm \Gamma }}_k = \bar \gamma _k - 2i{{\rm \Delta }}_k$. The Rabi frequencies Ω_*k*_ capture the variable dipole moments across the excited state manifold, including the optical selection rules. The last term in Eq. (3) corresponds to the Rydberg exciton interaction with the dipole–dipole matrix elements $V_{i{\prime}k,ij}^{\rm dd}\left( {\left| {{\bf{r{\prime}}} - {\bf{r}}} \right|} \right)$, coupling the product states $\left| {\phi _i,\phi _j} \right\rangle$ and $\left| {\phi _{i{\prime}},\phi _k} \right\rangle$. The polarizability

4 $${\cal P}({\bf{r}}) = \frac{i}{{{\rm \Gamma }}}\left( { - 2g{\cal E}({\bf{r}}) + \mathop {\sum}\limits_k {\kern 1pt} {{\rm \Omega }}_k\left\langle {\hat X_k({\bf{r}})} \right\rangle } \right)$$

can then be obtained from the steady-state expectation value of the Rydberg exciton coherence, $\left\langle {\hat X_k({\bf{r}})} \right\rangle$. While solving Eq. (3) generally poses an intractable quantum many-body problem, we seek an expansion in terms of the field amplitude ${\cal E}$, which allows us to obtain the polarizability in the form of Eq. (2). To this end, we consider the Heisenberg equations for $\hat X_k({\bf{r}})$, $\hat X_i^\dagger ({\bf{r}})\hat X_j({\bf{r}})$, $\hat X_i({\bf{r{\prime}}})\hat X_j({\bf{r}})$, $\hat X_i^\dagger ({\bf{r{\prime}}})\hat X_j({\bf{r}})$, and $\hat X_{i{\prime}}^\dagger ({\bf{r{\prime}}})\hat X_i({\bf{r{\prime}}})\hat X_j({\bf{r}})$ and discard all three-body terms in the resulting hierarchy of equations, since they do not contribute to the third-order nonlinearity *χ*^(3)^ in Eq. (2). Assuming an adiabatic steady state, for which $\partial _t\left\langle {\hat X_k({\bf{r}})} \right\rangle = 0$, $\partial _t\left\langle {\hat X_i^\dagger ({\bf{r}})\hat X_j({\bf{r}})} \right\rangle = 0$, etc., one thus obtains a closed set of algebraic equations that permits to express the Rydberg exciton coherence $\left\langle {\hat X_k({\bf{r}})} \right\rangle$ in terms of the photon field ${\cal E}$. The final crucial step is to appropriately transform from our product state basis, $\left| {\phi _i;\phi _j} \right\rangle$, to that of the interacting pair states, *μ*, which permits to establish the relation between $\left\langle {\hat X_k({\bf{r}})} \right\rangle$ and ${\cal E}$ in terms of the potential curves *U*_*μ*_ and the elements *c*_*ij*,*μ*_ (Supplementary Note 4). In this way, one can relate the rather complex manifold of interaction potential curves (Fig. 1c) to a single nonlocal nonlinear response function for the photon field (Fig. 1d).

Its real part defines an effective photon–photon interaction $W(r) \approx \frac{{{{\rm \Omega }}^2}}{{4g}}{{\rm Re}}\left[ {\chi ^{(3)}(r)} \right]$. As illustrated in Fig. 1d, the resulting nonlinear kernel is indeed highly nonlocal and extends over several hundred Bohr radii *a*_*b*_, much further than the characteristic length scale of interactions between ground state excitons of a few Bohr radii^10^. The obtained soft core shape of *W*(*r*) is consistent with the described blockade mechanism. Its strength can reach values up to several tenths of eV, which in part arises from the high excitonic fraction, ≈4*g*^2^/(4*g*^2^ + Ω^2^), of the dark-state polaritons formed under EIT conditions^13^. Remarkably, this behavior persists in a regime where the above simple picture of a single blockaded Rydberg state breaks down entirely and the broadened Rydberg exciton lines cover many interacting pair states that are shifted to near-resonant energies (Fig. 1c). This important effect can be traced back to the strong redistribution of the optical coupling strength, ${{\rm \Omega }} \to {\bar{\mathrm \Omega }}_\mu$, by the dipole–dipole interaction over a large number of interacting Rydberg pair states. This dilution of the Rabi coupling or, equivalently, the resulting strong broadening of the double excitation transition ultimately inhibits even near resonant excitation of interacting Rydberg excitons. Note that the height of the resulting soft core interaction

5 $$W(r \to 0) \approx \frac{{8g^2{{\rm \Delta \Omega }}^4}}{{\left| {{{\rm \Omega }}^2 + {{\rm \Gamma }}\bar \gamma } \right|^2 \cdot \left| {{\rm \Gamma }} \right|^2}}$$

is identical to the one estimated from the simplified single-state model as both mechanisms prevent the excitation of two nearby Rydberg excitons (Supplementary Note 5). It is this dilution blockade that facilitates the emergence of strong photon interactions even for substantially broadened Rydberg excitation lines, which is typically neglected for atomic systems but is fully accounted for in the present formalism.

Note that the extended range of the excitation blockade—either by shifting the pair state energy or diluting the corresponding optical coupling—justifies the applied treatment of the Rydberg exciton interactions. In fact, other mechanisms such as higher-order multipole couplings or exchange interactions would become relevant only at distances where a strong excitation blockade is established and, thus, do not contribute to the optical response (Supplementary Note 6).

**Fig. 2:**
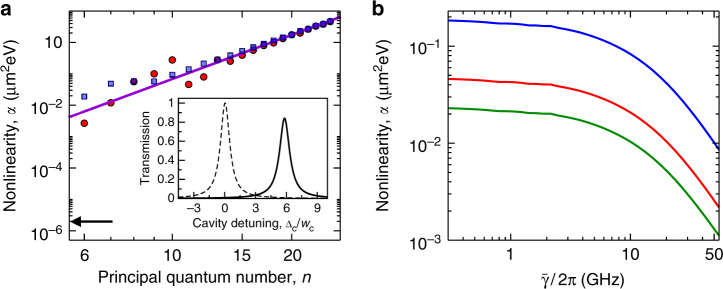
Optical nonlinearity induced by Rydberg interactions. **a** Integrated interaction strength *α* for the *m* = 1 excitonic series at the *K*^+^- (red) or *K*^−^-point (blue) of the Brillouin zone with Ω/2*π* = 60 GHz, $\bar \gamma = \gamma {{\rm /}}n^3$ and the remaining parameters as in Fig. 1, i.e., *g*/2*π* = 5561 GHz^31^, Δ/2*π* = 700 GHz, and *γ*/2*π* = 300 GHz. The solid line shows the *α* ~ *n*^14/3^ scaling found for both *K*-points which results from the large-distance ~*n*^11^ dependence of the Rydberg exciton interaction and the considered $\bar \gamma \sim n^{ - 3}$ decrease of their linewidth^12^. For lower principal quantum numbers, however, the interaction strength features a significant valley dependence. For comparison, the arrow indicates the nonlinearity measured^10^ for ground-state excitons in GaAs. The inset shows corresponding transmission spectra upon changing the cavity frequency for *n* = 10 in the linear regime (dashed line) and for small driving $\left| {\cal E} \right|^2 = 2.5 \times 10^{ - 5}$ μm^−2^ (solid line). Both curves are scaled by the maximum of the linear transmission line. The cavity detuning Δ_c_ is shown in units of the width *w*_c_ of the linear transmission line. **b** illustrates that the nonlinearity persists in the presence of significant Rydberg state broadening, $\bar \gamma$, as shown for *n* = 10, *γ*/2*π* = 300 GHz (blue), *γ*/2*π* = 1200 GHz (red) and *γ*/2*π* = 2400 GHz (green) with Δ = 2*γ* and at constant $\frac{{{{\rm \Omega }}^2}}{{\left| {{\rm \Gamma }} \right|}} = 40$ GHz

The nonlinearity can be probed experimentally by measuring the associated frequency shift $\delta _{{{\rm nl}}} \approx \frac{{4g^2}}{{{{\rm \Omega }}^2}}\alpha \left| {\cal E} \right|^2$ of the cavity transmission line. Here *α* = ∫d*r*^2^*W*(*r*) characterizes the effective photon interaction, in equivalence to the effective polariton scattering length arising from collisional exciton interactions as observed, for example, in GaAs^10^. As shown in Fig. 2a, the interaction strength achievable with Rydberg excitons exceeds that of collisional interactions by several orders of magnitude and it strongly increases with the principal quantum number $\sim n^{\frac{{14}}{3}}$.

While the finite Rydberg state linewidth can often be neglected for atomic systems^15^, it may present a major limiting factor for solid-state settings. However, as shown Fig. 2b, the vast enhancement of the optical nonlinearity even persists for a substantial broadening of $\bar \gamma \approx 0.1$ meV, which is one order of magnitude higher than what would be expected from pure radiative decay in WSe_2_ monolayers^29^. Equally important, the enormous strength of the nonlinearity makes it possible to operate at such low probe-light intensities that additional exciton-density dependent effects^29,30^ would not degrade the coherence of the system in the present situation.

### Photon statistics

**Fig. 3:**
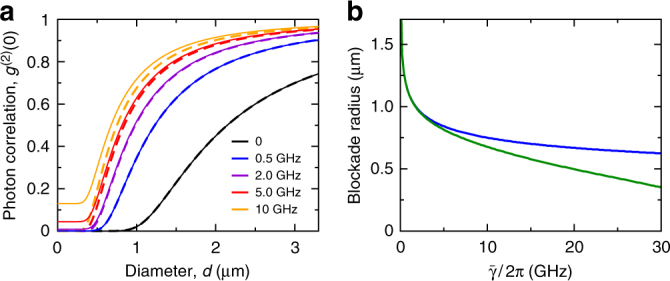
Photon correlations. **a** Equal-time photon-photon correlations *g*^(2)^(0) of the transmitted light as a function of the diameter, *d*, of the illumination area for different Rydberg state linewidths, $\bar \gamma {{\rm /}}2\pi$, and resonant excitation of the 10*p*-exciton at K^+^. **b** Critical photon-blockade radius $R_{{\rm b}}^{({{\rm ph}})}$ for which *g*^(2)^(0) crosses 0.5 (green) as a function of $\bar \gamma$ at fixed $\frac{{{{\rm \Omega }}^2}}{{\left| {{\rm \Gamma }} \right|}} = 40$ GHz. The blue line shows the corresponding size $R_{{\rm b}}^{({{\rm X}})}$ at which the probability to excite two Rydberg excitons within the illumination area is suppressed by a factor of 2. This exciton blockade radius scales as $\sim \bar \gamma ^{ - 1/6}$

Having established the emergence of a strong nonlinear response to weak coherent light fields in spatially extended geometries, we can finally explore its effect on a few-photon quantum level. To this end, we now consider the limit of a cavity that is coherently driven over a small illumination area with diameter *d*. For sufficiently small *d*, the cavity can only accommodate a single Rydberg exciton due to either or both of the blockade mechanisms described above. As a result, a single cavity polariton will expose the high optical response of the low-lying exciton transition and it is thus expected to ultimately block photon transmission for a sufficiently strong cavity coupling. To describe this effect we now start from the Heisenberg equation for the photon field operator $\hat {\cal E}$ and use its adiabatic solution

6 $$\hat {\cal E}({\bf{r}},t) = \frac{{2g}}{{{{\rm \Gamma }}_{{{\rm cav}}}{{\rm \Gamma }} + 4g^2}}\mathop {\sum}\limits_k {\kern 1pt} {{\rm \Omega }}_k\hat X_k({\bf{r}},t) + \frac{{2{{\rm \Gamma }}\eta }}{{{{\rm \Gamma }}_{{{\rm cav}}}{{\rm \Gamma }} + 4g^2}}E^{{{\rm in}}}$$

to re-express the photon field in terms of the Rydberg exciton operators. Here Γ_cav_ = *κ* − 2*i*Δ_c_ is determined by the cavity decay constant *κ* and the cavity detuning Δ_c_ and *E*_in_ is the strength of the coherent cavity driving field while *η* denotes the corresponding efficiency of the photon cavity coupling. This yields a closed equation for the exciton dynamics which can be solved and used to analyze the photon statistics using Eq. (6) (Supplementary Note 7). Figure 3a shows the two-photon correlation correlation function at zero time delay, *τ*,

7 $$g^{(2)}(\tau = 0) = \left[ {\pi (d{{\rm /}}2)^2} \right]^{ - 2}{\int}_D {\kern 1pt} {{\rm d}}{\bf{r}}_1 {\int}_D {\kern 1pt} {{\rm d}}{\bf{r}}_2\frac{{\left\langle {\hat {\cal E}^\dagger \left( {{\bf{r}}_1,t} \right)\hat {\cal E}^\dagger \left( {{\bf{r}}_2,t} \right)\hat {\cal E}\left( {{\bf{r}}_2,t} \right)\hat {\cal E}\left( {{\bf{r}}_1,t} \right)} \right\rangle }}{{\left\langle {\hat {\cal E}^\dagger \left( {{\bf{r}}_1,t} \right)\hat {\cal E}\left( {{\bf{r}}_1,t} \right)} \right\rangle \left\langle {\hat {\cal E}^\dagger \left( {{\bf{r}}_2,t} \right)\hat {\cal E}\left( {{\bf{r}}_2,t} \right)} \right\rangle }}$$

integrated over the illumination area *D* of size *π*(*d*/2)^2^. Indeed, the effective photon-photon interactions are found to be strong enough to alter the photon statistics over rather large distances and generate nonclassical states with *g*^(2)^(0) < 1 for up to *d* ~ 1 μm. The collectively enhanced cavity-coupling to the spatially extended illumination area even enables single-photon transmission with *g*^(2)^(0) ≈ 0. For small values of *d*, the achievable photon correlations are limited by the Rydberg exciton linewidth according to

8 $$g^{(2)}(0) \approx 4\frac{{\bar \gamma ^2\left| {{\rm \Gamma }} \right|^2}}{{{{\rm \Omega }}^4}},$$

if $\bar \gamma < {{\rm \Omega }}^2{{\rm /}}\left| {{\rm \Gamma }} \right|$ and *g*^2^ ≫ *γκ*. Importantly, however, the exciton blockade is largely unaffected by Rydberg state decay and decoherence, with a blockade radius that decreases only weakly as $R_{{\rm b}}^{({{\rm X}})}\sim \bar \gamma ^{ - 1/6}$ and remains on a *μ*m scale even as $\bar \gamma$ approaches the EIT linewidth ${{\rm \Omega }}^2{{\rm /}}\left| {{\rm \Gamma }} \right|$ (Fig. 3b). For Cu_2_O^12^, the stronger interactions and longer Rydberg state lifetimes suggest even larger interaction ranges of $R_{{\rm b}}^{({{\rm X}})} \approx 3.1$ μm for *n* = 10 and $R_{{\rm b}}^{({{\rm X}})} \approx 14.6$ μm for *n* = 20 in an equivalent setup.

## Discussion

Our results show that Wannier Rydberg excitons provide an intrinsic mesoscopic lengthscale capable of supporting collective excitations that permit the all-optical manipulation of light. Yet, this photon interaction range can be much smaller than typical system sizes, which opens up a new regime of photonic many-body physics, well beyond the capabilities of corresponding atomic systems. With Cu_2_O and TMDC monolayers two promising and complementary platforms featuring Rydberg excitations have emerged. From an optics perspective, a great advantage of single-layered TMDCs is the strong achievable light-matter coupling and two-dimensional geometry, which is naturally suited for integration in microcavities^31^. A central challenge lies in the exciton linewidth that depends on several factors, such as the temperature, exciton density, and the addressed states. Recent experiments^32–34^ on ground state excitons in TMDC monolayers have achieved a strong suppression of inhomogeneous broadening effects well below the homogenous linewidth, which at low temperatures and exciton densities predominantly arises from radiative decay, with measured values of *γ*/2*π* ~ 400 GHz^29^. We have shown that the nonlinear phenomena discussed in this work persist even at ten times larger linewidths than estimated from the expected ~ *n*^−3^ scaling of the rate of radiative Rydberg state decay. We finally note that increasing the control field strength Ω would allow to tolerate yet broader Rydberg state linewidths due to an enlarged EIT window, whilst only leading to a minor decrease of the blockade radius.

Experiments with Cu_2_O have made it possible to observe the $\bar \gamma \propto n^{ - 3}$ scaling of the linewidth, where Rydberg state excitation to quantum numbers of *n* = 25 could be demonstrated^12^. While the *np*-Rydberg series probed in these experiments appears behind a broad phonon background originating from the excitation of $\left| {1s} \right\rangle$ excitons together with LO phonons at around 13.2 meV^35^, the two-photon coupling to $\left| {ns} \right\rangle$-Rydberg excitons^36^ considered in this work suggests strongly suppressed direct phonon-generation and significantly improved coherence properties since the $\left| {2p} \right\rangle$-population is strongly suppressed under EIT conditions. An open challenge for Cu_2_O excitons is the construction of efficient cavities to compensate for the otherwise weak (dipole-forbidden) exciton coupling constant.

The exaggerated properties of Rydberg excitons^37^ combined with the integrability and continually advancing functionalities of low-dimensional and bulk semiconductors^38,39^ hold promise for few-photon^9^ applications in both systems. Already on a classical level, the typical time and energy scales of the described systems may enable fast optical switching at ultralow light-intensities, and the exploration of new collective nonlinear phenomena in exciton-polariton condensates.

### Data availability

All data generated in this study are available from the corresponding author upon request.

## Electronic supplementary material

Supplementary Information(PDF 8739 kb)
