# Supplementary material for: Giant optical nonlinearities from Rydberg excitons in semiconductor microcavities
Source: Nat Commun. 2018 Apr 3;9:1309. doi: 10.1038/s41467-018-03742-7 (PMC5883042; doi:10.1038/s41467-018-03742-7)
Supplement: Supplementary file 1 — Supplementary Information(PDF 8739 kb) [file 41467_2018_3742_MOESM1_ESM.pdf]

Supplementary Information:
Giant optical nonlinearities from Rydberg excitons in semiconductor microcavities

Walther et al.

Supplementary Note 1: Single Exciton Solution

Semiconductor excitons are bound electron hole pairs whose binding energy reduces the free electron band gap to the quasi-particle band gap, which is important and in some cases dominant for the optical properties. Both electron and hole have intricate dispersion relations inherent to the material's band structure. Transition metal dichalcogenide (TMDCs) are direct semiconductors in the monolayer limit with least energy transitions at the ±K-points, the corners of the hexagonal Brillouin zone [1]. Though monolayer TMDCs resemble graphene in many ways, their band structures differ in (at least) one mayor feature: While graphene exhibits Dirac cones at the K-points, the valence and conduction bands in TMDCs are split naturally by a sizable band gap, rendering the dispersions quadratic for small reciprocal vectors $\mathbf{k}$ off the K-points. Excitons in TMDCs are formed at the K-points and have a large binding energy on the order of 0.5 meV, making them stable against thermal fluctuations even at room temperature. A full theoretical description requires ab-initio methods, but an accurate and very insightful description is given by the standard effective model [2, 3]

$$2E_{\mathbf{k}}f(\mathbf{k}) + \sum_{\mathbf{k}'} f(\mathbf{k}')V_{\mathbf{k},\mathbf{k}'} \langle c\mathbf{k} \,|\, c\mathbf{k}'\rangle \langle v\mathbf{k} \,|\, v\mathbf{k}'\rangle = Ef(\mathbf{k}), \tag{1}$$

where $E_{\mathbf{k}}$ is the electron/hole dispersion, $f(\mathbf{k})$ are the amplitudes of creating a exciton at relative wavevector $\mathbf{k}$ and $V_{\mathbf{k},\mathbf{k}'}$ are the Fourier components of the electron-hole potential $V_{\mathrm{eh}}(r)$. The Bloch states overlaps $\langle c\mathbf{k} \,|\, c\mathbf{k}'\rangle$ (conduction band) and $\langle v\mathbf{k} \,|\, v\mathbf{k}'\rangle$ (valence band) imprint the crystal topology onto the excitons. In an expansion around $\pm K$ these overlaps can be expressed as $\mathbf{k}$ dependent functions of the Berry curvature Ω_0 with the real space analogs [3]

$$V = V_{\mathrm{eh}}(r) + \frac{-\tau|\Omega_0|}{2\hbar}(\nabla V_{\mathrm{eh}} \times \mathbf{p})_z + \frac{|\Omega_0|}{4}\nabla^2 V_{\mathrm{eh}}(r) \tag{2}$$

where $\tau = \pm 1$ denotes the valley index and $\mathbf{p}$ is the momentum operator. Monolayer TMDCs being virtually two-dimensional (2d) materials, the interesting situation arises in which the excitonic wavefunction is confined to the plane, while electromagnetic interactions can also enter the surrounding environment, i.e. are three-dimensional (3d). As a result, the potential is screened at short separations between electron and hole (as is typical in bulk materials), but essentially unscreened at larger distances. Following the model in [4–9] of this situation we use the well-established potential

$$V_{\mathrm{eh}}(r) = -\frac{e^2}{4\pi\epsilon_0}\frac{\pi}{2r_0}\left[H_0(r/r_0) - Y_0(r/r_0)\right], \tag{3}$$

where H_0 is the first Struve function and Y_0 is first Bessel function of the second kind and r_0 is the effective screening length (taken from [6]), capturing the cross-over between Coulomb behavior at large r and a (weaker) logarithmic decay at small r. Introducing center-of-mass and relative coordinates

$$\mathbf{R}_i = \frac{m_{\mathrm{e}}\mathbf{r}_{\mathrm{e}i} + m_{\mathrm{h}}\mathbf{r}_{\mathrm{h}i}}{m_{\mathrm{e}} + m_{\mathrm{h}}} \tag{4}$$

$$\mathbf{r}_i = \mathbf{r}_{\mathrm{e}i} - \mathbf{r}_{\mathrm{h}i}. \tag{5}$$

the Schrödinger equation for the relative coordinate reads

$$-\frac{\hbar^2}{2\mu}\Delta\psi - (E - V(r))\,\psi = 0, \tag{6}$$

where $\mu = \frac{m_{\mathrm{e}}m_{\mathrm{h}}}{m_{\mathrm{e}}+m_{\mathrm{h}}}$ is the reduced mass. A product ansatz $\psi(\mathbf{r}) = \rho(r)\Phi(\phi)$ gives

$$-\frac{\hbar^2}{2\mu}\left[\frac{r^2}{\rho}\frac{\partial^2\rho}{\partial r^2} + \frac{r}{\rho}\frac{\partial\rho}{\partial r} + \frac{1}{\Phi}\frac{\partial^2\Phi}{\partial\phi^2}\right] - (E - V(r))r^2 = 0, \tag{7}$$

where we refer to m as the "angular" quantum number (cf. below for comparison with 3d case), leading directly to the orbital eigenfunctions

$$\Phi(\phi) = \frac{1}{\sqrt{2\pi}}e^{im\phi}, \; m \in \mathbb{Z}. \tag{8}$$

The radial equation then reads

$$\frac{d^2\rho}{dr^2} + \frac{1}{r}\frac{d\rho}{dr} + \left[\frac{2\mu}{\hbar^2}\left(E - V(r)\right) - \frac{m^2}{r^2}\right]\rho = 0. \tag{9}$$

We cast the radial solutions as $u(r) = r^\beta \rho(r)$, transforming the radial equation into

$$\frac{d^2u}{dr^2} = \left[-\frac{2\mu}{\hbar^2}(E - V(r)) + \frac{m^2 - \beta^2}{r^2}\right]u(r) + \frac{2\beta - 1}{r}\frac{du(r)}{dr}. \tag{10}$$

Standard numerical methods work fine for $m > 0$ when the term $(m^2 - \beta^2)/r^2$ constitutes an effective repulsive potential. However, for $m = 0$ this term becomes attractive and at small distances it dominates the Coulomb potential $-\beta^2/r^2 \ll -1/r$ if $0 < r < \delta$. The resulting equation is that of a one-dimensional Schrödinger equation with a potential $V(\rho) \propto 1/\rho^2$. This potential has very unusual properties [10] because it is just on the boundary of permitting bound states (those are impossible for stronger potentials $V \propto 1/r^{2+\epsilon}$). We follow a recently proposed algorithm [11] which suggests an Euler integrator anticipating the correct solution.

Let us now comment on the physical implications of this exciton model: The excitonic wavefunction is fully characterized by the the principal quantum number n determined from the radial solution and and the angular part m. Compared to the 3d Coulomb case, the quantum number l is "frozen" at its maximum value, thus leaving only n and m [12]. The latter determines the optical properties, such that we refer to $m = 0$ as s-states, $|m| = 1$ as p-states etc. While the exact $1/r$ Coulomb potential leads to a complete degeneracy of all states at given n, the screened potential V_{eh} splits the energy states according to $|m|$. Contrary to expectations based on the 3d counterpart, this (quantum) defect scales almost linearly with $|m|$, allowing to optically address states other than the s-states (Supplementary Figure 1). The energy shifts are quite pronounced and dominate the non-hydrogenic nature of the spectrum [6], whereas the second and third terms in Supplementary Equation 2 are small corrections. They are topological terms, accounting for a combination of strong spin-orbit coupling in TMDCs and the breaking of inversion symmetry which results in the six K-points falling into two different classes ±K [13], characterized by Berry curvatures $\pm\Omega_0$. While both Berry terms are rotationally symmetric and, thus, do not mix (bare) states of different m, the first term acts like a position dependent magnetic field $(\nabla V \times \mathbf{p})_z = B(r)L_z, B(r) = |\nabla V|$, lifting the degeneracy in $\pm m$. Note that this breaking of time reversal symmetry is opposite at ±K, restoring overall time reversal symmetry. This simplified expansion around K was first proposed for $n = 2$ where the energy splitting is largest [2]. We emphasize that the approximation improves as higher quantum numbers are considered.

TMDCs feature valley spin-orbit coupling of the valence and (to a lesser extent) the conduction band, such that the bands acquire different energy shifts and are susceptible to either σ^+ or σ^- light [13]. By using either of the circularly polarized types of light, we can restrict our attention to the quadratic regions close the +K or -K points in the Brillouin zone. For the intra-excitonic transitions to the Rydberg states optical selection rules arise directly from the excitonic wavefunctions: only transition with $m' = m \pm 1$ are dipole-allowed. In particular, we can select $m' = m + 1$ using σ^+ light and $m' = m - 1$ by using σ^- light.

Supplementary Note 2: Excitonic Pair Interactions

Excitonic ground state interactions are typically of exchange character: The wavefunctions of two excitons overlap giving rise to interactions based on the fermionic nature of the electrons. If addressing Rydberg states, excitons are usually separated by hundreds of Bohr radii, such that the primary type of interaction is electromagnetic. At such great distances r screening effects become negligible for the aforementioned reasons

$$\lim_{r/r_0 \gg 1} V_{\mathrm{eh}}(r) \to -\frac{e^2}{4\pi\epsilon_0}\frac{1}{r}\left[1 + \mathcal{O}((r/r_0)^{-2})\right] \tag{11}$$

and the resulting Coulombic terms

$$V^{\mathrm{dd}} = \frac{e^2}{4\pi\epsilon_0}\left(-\frac{1}{r_{\mathrm{h_1e_2}}} - \frac{1}{r_{\mathrm{e_1h_2}}} + \frac{1}{r_{\mathrm{h_1h_2}}} + \frac{1}{r_{\mathrm{e_1e_2}}}\right), \tag{12}$$

where $r_{\mathrm{h_1e_2}}$ is the separation of the first exciton's electron from the second exciton's hole and so on, are expanded into a standard van der Waals potential with respect to the center of mass coordinates, using expansions of the form

$$\frac{1}{r_{\mathrm{h_1e_2}}} \approx \frac{1}{R}\left(1 - \frac{1}{MR}q^z_{\mathrm{e_1h_2}} - \frac{1}{2}\left[\frac{1}{RM}\mathbf{q}_{\mathrm{e_1h_2}}\right]^2 + \frac{3}{8}\left[\frac{2}{MR}q^z_{\mathrm{e_1h_2}}\right]^2\right), \tag{13}$$

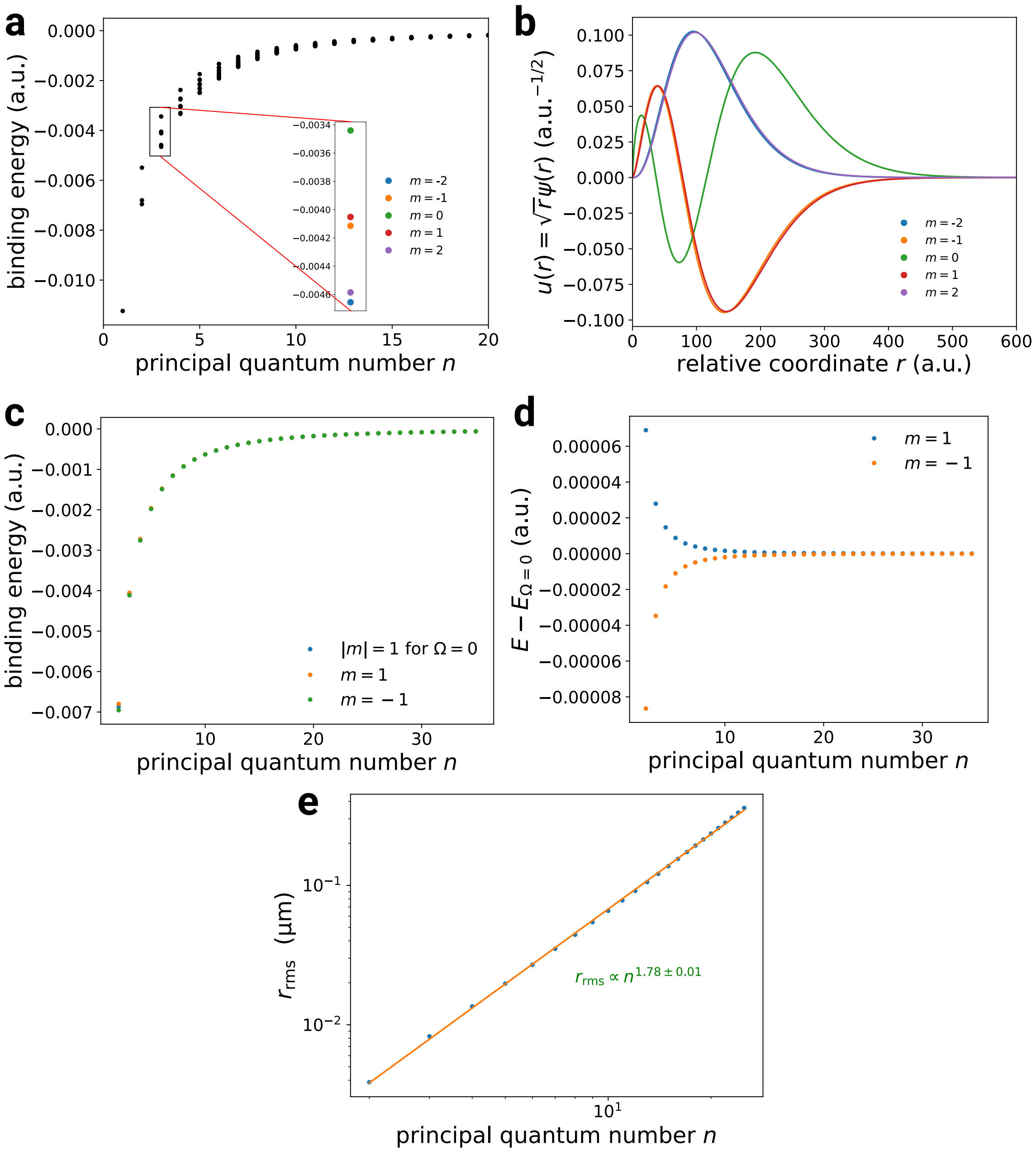

Supplementary Figure 1: Overview of single exciton states. (a) The excitonic spectrum at K^+ is described by quantum numbers n and m. Due to screening and Berry curvature there are no degenerate states (inset a) and slight differences in the wavefunctions of the two p-states appear (b). (c) For example, Berry curvature effects split these p-states (green and orange), leading to a deviation from the spectrum without Berry curvature (blue). (d) This splitting decreases rapidly with increasing n. (e) The root-mean-square separation between electron and hole scales like $n^{1.78\pm0.01}$, where the standard deviation was obtained from a least-squares fit to the numerical data.

where $\mathbf{R} = \mathbf{R}_1 - \mathbf{R}_2$ is the vector connecting the excitons' centers of mass, $\mathbf{q}_{\mathrm{e}_1\mathrm{h}_2} = m_{\mathrm{h}}\mathbf{r}_1 + m_{\mathrm{e}}\mathbf{r}_2$ and $q^z_{\mathrm{e}_1\mathrm{h}_2} = \mathbf{q}_{\mathrm{e}_1\mathrm{h}_2}\mathbf{R}/R$ is the projection of this vector on $\mathbf{R}$. We arrive at the well-known dipole-dipole interaction (in relative and center of mass coordinates) with $z_i = \mathbf{r}_i\mathbf{R}/R$

$$V^{\mathrm{dd}} = \frac{e^2}{4\pi\epsilon_0}\frac{1}{M^2R^3}\left[M^2\mathbf{r}_1\mathbf{r}_2 - 3M^2 z_1 z_2\right] = \frac{e^2}{4\pi\epsilon_0}\frac{1}{R^3}\left[\mathbf{r}_1\mathbf{r}_2 - 3z_1z_2\right]. \quad (14)$$

The excitonic pair Hamiltonian

$$\hat{H} = \hat{H}_1^{\mathrm{ex}} + \hat{H}_2^{\mathrm{ex}} + \hat{V}^{\mathrm{dd}} \tag{15}$$

acts on the Hilbert space spanned by the product states $\{|\alpha\rangle = |\psi_{n_1 m_1};\psi_{n_2 m_2}\rangle \equiv |\psi_i,\psi_j\rangle\}$. This basis diagonalizes the single-particle contributions for the first exciton

$$\hat{H}_1^{\mathrm{ex}} = \sum_{n,m} E_{n,m} \left|\psi_{n,m}(\mathbf{r}_1)\right\rangle \left\langle\psi_{n,m}(\mathbf{r}_1)\right| \otimes \mathbb{1}, \tag{16}$$

where $E_{n,m}$ is the energy of state $|n,m\rangle$, and likewise for the second exciton but it is coupled by the off-diagonal terms in

$$V^{\mathrm{dd}} = \frac{V^{\mathrm{dd,\,rad}}}{4}\left[-(\delta_{m_1,m_1'+1}\delta_{m_2+1,m_2'} + \delta_{m_1+1,m_1'}\delta_{m_2,m_2'+1}) - 3(e^{-2i\theta}\delta_{m_1,m_1'+1}\delta_{m_2,m_2'+1} + e^{2i\theta}\delta_{m_1+1,m_1'}\delta_{m_2+1,m_2'})\right], \tag{17}$$

$$V^{\mathrm{dd,\,rad}} = \left\langle \rho_{n_1 m_1}(\mathbf{r}_1) \,\middle|\, \mathbf{r}_1 \,\middle|\, \rho_{n_1' m_1'}(\mathbf{r}_1)\right\rangle \cdot \left\langle \rho_{n_2 m_2}(\mathbf{r}_2) \,\middle|\, \mathbf{r}_2 \,\middle|\, \rho_{n_2' m_2'}(\mathbf{r}_2)\right\rangle, \tag{18}$$

where we can w.l.o.g. choose the absolute in-plane orientation $\theta = 0$, $V^{\mathrm{dd,\,rad}}$ falls off with R^{-3} and contains the dipole matrix elements. The numerical diagonalization is facilitated by the symmetries of the excitonic Hamiltonian. First, as is obvious from the pair basis representation, V^{dd} does not couple states with even and odd $M = m_1 + m_2$. Second, the entire Hamiltonian is invariant w.r.t. particle exchange, implying that the Hilbert space can be decomposed into non-interacting subspaces of odd and even states. In a LCAO-type numerical procedure, we diagonalize the excitonic spectrum in the subspace of symmetric states with even M with a LAPACK algorithm for sparse matrices, ensuring convergence by varying the basis set.

By diagonalizing the Hamiltonian from Supplementary Equation 15 we obtain the excitonic interaction potential surfaces U_μ, as shown for example in Fig. 1c in the main text. As the van der Waals interaction increase enormously with n, it couples only excited states and leaves the excitonic ground states unchanged. Thus, we summarize the diagonalization formally in the unitary transformation

$$|\mu\rangle = \hat{U}^\dagger |\alpha\rangle = \sum_\alpha c^*_{\alpha,\mu} |\alpha\rangle, \tag{19}$$

restricting $|\alpha\rangle$ to the manifold of doubly excited states.

Supplementary Note 3: Total Hamiltonian and Equations of Motion

We consider an infinite system of excitons in a cavity. Each exciton has a vacuum state, an excitonic ground state g (corresponding to $|1s\rangle$ and created by $\hat{X}_g^\dagger$) at $\hbar\omega_g^{\mathrm{ex}}$ and a number of interacting Rydberg states $\{k\}$ (corresponding to $|np\rangle$ and created by $\hat{X}_k^\dagger$) at energies $\hbar\omega_k^{\mathrm{ex}}$. The excitons are driven by a weak cavity field (operator $\hat{\mathcal{E}}$) at frequency ω_{p} and a strong external laser field. We consider a single longitudinal cavity mode (for simplicity assume the lowest energy mode), such that we can describe the in-plane light propagation via a Schrödinger equation with effective mass $m_{\mathrm{ph}} = \hbar n k_z/c$ [14], where $k_z = \frac{\pi}{l_z}$, l_z is the cavity width, n is the dielectric constant in the cavity and c is the vacuum speed of light. This picture is valid if $k/k_z \ll 1$ (quadratic band) and $\frac{\hbar^2 k^2}{2m} \ll \frac{2\pi\hbar c}{n l_z} \leftrightarrow k \ll \frac{2\pi}{l_z}$ (large separation of odd modes), which is (practically) equivalent to the first condition. As is standard in the low-excitation regime (discussed in detail below), we bosonize the exciton operators, such that the Hamiltonian for the case of a coherent driving field

$\hat{E}^{\rm in}(\mathbf{r},t) = \hat{E}_0^{\rm in} e^{-i\omega_{\rm in} t}$ with coupling constant η reads, in rotating wave approximation $\hat{H} = \int d^2\mathbf{r}\hat{h}(\mathbf{r})$ with

$$\begin{aligned}
\hat{h}(\mathbf{r}) &= \hat{\mathcal{E}}(\mathbf{r}) \left(-\frac{\hbar^2}{2m_{\rm ph}} \nabla^2 + \hbar\omega_{\rm cav} \right) \hat{\mathcal{E}}^\dagger(\mathbf{r}) \\
&+ \hat{X}_g^\dagger(\mathbf{r}) \left(-\frac{\hbar^2}{2m_{\rm ex}} \nabla^2 + \hbar\omega_g^{\rm ex} \right) \hat{X}_g(\mathbf{r}) \\
&+ \sum_i \hat{X}_i^\dagger(\mathbf{r}) \left(-\frac{\hbar^2}{2m_{\rm ex}} \nabla^2 + \hbar\omega_i^{\rm ex} \right) \hat{X}_i(\mathbf{r}) \\
&+ \hbar g \left(\hat{\mathcal{E}}^\dagger(\mathbf{r}) \hat{X}_g(\mathbf{r}) + h.c. \right) - \sum_i \hbar \frac{\Omega_i}{2} \left(\hat{X}_g^\dagger(\mathbf{r}) \hat{X}_i(\mathbf{r}) + h.c. \right) \\
&+ \sum_{i \le j, i' \le j'} \int d\mathbf{r}' \hat{X}_{i'}^\dagger(\mathbf{r}) \hat{X}_{j'}^\dagger(\mathbf{r}') V^{\rm dd}_{i'j',ij}(|\mathbf{r}-\mathbf{r}'|) \hat{X}_i(\mathbf{r}) \hat{X}_j(\mathbf{r}') \\
&+ i\hat{\mathcal{E}}^\dagger(\mathbf{r}) \eta \hat{E}(\mathbf{r}) + h.c.,
\end{aligned} \tag{20}$$

where $\hat{\mathcal{E}}(\mathbf{r})$ and $\hat{X}_g(\mathbf{r})$ rotate at $e^{i\omega_{\rm in} t}$ with respect to the lab frame, while $\hat{X}_k$ rotates at $e^{i(\omega_{\rm in}+\omega_{\rm c})t}$ relative to the lab frame. Summarizing the previous Supplementary Notes, the first, second and third terms represent the free in-plane motion of the cavity field, the ground state and excited state excitons, respectively. The fourth line contains the laser coupling between vacuum and ground state excitons as well as the subsequent excitation to Rydberg levels (at driving frequency $\omega_{\rm c}$), while the second to last line describes dipole coupling between pairs of excitons, inducing many-body correlations. Balancing the pumping (final line), the excitonic dynamics is naturally subject to decoherence processes: Excited states may spontaneously decay to lower and dipole coupled states, but there are also dephasing mechanisms attenuating quantum coherences. Assuming δ-correlated decoherence mechanisms, we model both types of processes by single-particle Lindblad master equations $\mathcal{L}(\rho) = \hat{L}^\dagger \rho \hat{L} + \frac{1}{2}\left(\hat{L}^\dagger \hat{L} \rho + \rho \hat{L}^\dagger \hat{L} \right)$. In the limit of very small excitation fractions decay of excitons can be modeled as dephasing. Additionally, photons may leak out of the semiconductor cavity through imperfect mirrors at a loss rate κ. Within the used approximations, we can use an effective Hamiltonian to formulate Heisenberg equations of motion

$$\partial_t \hat{\mathcal{E}}(\mathbf{r}) = i\omega_{\rm in} \hat{\mathcal{E}}(\mathbf{r}) - i\left(\omega_{\rm cav} - \frac{\hbar}{2m_{\rm ph}} \nabla^2 \right) \hat{\mathcal{E}}(\mathbf{r}) - ig\hat{X}_g(\mathbf{r}) - \frac{\kappa}{2} \hat{\mathcal{E}}(\mathbf{r}) + \eta \hat{E}^{\rm in}(\mathbf{r}) \tag{21}$$

$$\partial_t \hat{X}_g(\mathbf{r}) = i\omega_{\rm in} \hat{X}_g(\mathbf{r}) - ig\hat{\mathcal{E}}(\mathbf{r}) - i\left(-\frac{\hbar}{2m_{\rm ex}} \nabla^2 + \omega_g^{\rm ex} \right) \hat{X}_g(\mathbf{r}) + i\sum_i \frac{\Omega_i}{2} \hat{X}_i(\mathbf{r}) - \frac{\gamma}{2} \hat{X}_g(\mathbf{r}) \tag{22}$$

$$\begin{aligned}
\partial_t \hat{X}_k(\mathbf{r}) &= i(\omega_{\rm in} + \omega_c) \hat{X}_k(\mathbf{r}) + i\frac{\Omega_k}{2} \hat{X}_g(\mathbf{r}) - i\left(-\frac{\hbar}{2m_{ex}} \nabla^2 + \omega_k^{\rm ex} \right) \hat{X}_k(\mathbf{r}) \\
&- i\sum_{i \le j, i'} \int d^2r' \hat{X}_{i'}^\dagger(\mathbf{r}') \frac{V^{\rm dd}_{i'k,ij}(|\mathbf{r}'-\mathbf{r}|)}{\hbar} \hat{X}_i(\mathbf{r}') \hat{X}_j(\mathbf{r}) - \frac{\bar{\gamma}_k}{2} \hat{X}_k(\mathbf{r}).
\end{aligned} \tag{23}$$

We introduce the following notation

$$\Delta_{\rm c} \equiv \omega_{\rm in} - \omega_{\rm cav} \qquad \Gamma_{\rm cav} \equiv \kappa - i2\Delta_{\rm c} \tag{24}$$

$$\Delta \equiv \omega_{\rm in} - \omega_g^{\rm ex} \qquad \Gamma \equiv \gamma - i2\Delta \tag{25}$$

$$\Delta_k \equiv \omega_{\rm in} + \omega_{\rm c} - \omega_k^{\rm ex} \qquad \Gamma_k \equiv \bar{\gamma}_k - i2\Delta_k. \tag{26}$$

and eliminate the intermediate state

$$\hat{X}_g(\mathbf{r}) = 2i \frac{-2g\hat{\mathcal{E}}(\mathbf{r}) + \sum_i \Omega_i \hat{X}_i(\mathbf{r})}{2\Gamma}. \tag{27}$$

Here and in the following we neglect the excitonic dispersion, which is valid if the emergent structures are larger than $\lambda_{\rm c} \gtrsim 2\pi\sqrt{2m_{\rm ex}\delta_{\rm EIT}/\hbar}^{-1}$ with $\delta_{\rm EIT} = \Omega^2/|\Gamma|$ and the polariton is sufficiently photon-like $\frac{m_{\rm ph}}{m_{\rm ex}} \ll \left(\frac{\Omega}{2g}\right)^2$, as is satisfied for typical cavity parameters. We further remark that the adiabatic elimination of Supplementary Equation 27 is

exact (in the steady state) only for non-interacting excitons. The approximation we make by taking it over to the interacting many-body system works well if $\Omega \ll \sqrt{\Delta^2 + \gamma^2}$ [15]. Note that this is last step reduces complexity from the equations but it is no fundamental limitation, as the intermediate state can be treated fully. The final equations of motion, as given in the main text, read

$$\partial_t \hat{\mathcal{E}}(\mathbf{r}) = -\frac{\Gamma_{\text{cav}}}{2}\hat{\mathcal{E}}(\mathbf{r}) + i\frac{\hbar}{2m_{\text{ph}}}\nabla^2\hat{\mathcal{E}}(\mathbf{r}) - \frac{2g^2}{\Gamma}\hat{\mathcal{E}}(\mathbf{r}) + \frac{g}{\Gamma}\sum_i \Omega_i \hat{X}_i(\mathbf{r}) + \eta\hat{E}^{\text{in}}(\mathbf{r}) \tag{28}$$

$$\partial_t \hat{X}_k(\mathbf{r}) = \frac{g\Omega_k}{\Gamma}\hat{\mathcal{E}}(\mathbf{r}) - \frac{\Gamma_k}{2}\hat{X}_k(\mathbf{r}) - \frac{\Omega_k}{2\Gamma}\sum_i \Omega_i \hat{X}_i(\mathbf{r}) - i\sum_{i\leq j,i'}\int d\mathbf{r}'\hat{X}^\dagger_{i'}(\mathbf{r}')\frac{V^{\text{dd}}_{i'k,ij}(|\mathbf{r}'-\mathbf{r}|)}{\hbar}\hat{X}_i(\mathbf{r}')\hat{X}_j(\mathbf{r}). \tag{29}$$

These equations fully specify the temporal evolution of the quantum many-body system and are the starting point of all further calculations.

Supplementary Note 4: Nonlinear Optical Response

As stated in the main text, we solve the many problem of Supplementary Equation 29 to leading order in the coherent photon field $\mathcal{E}(\mathbf{r}) = \langle\hat{\mathcal{E}}(\mathbf{r})\rangle$. For convenient numerical diagonalization we introduce the interaction Hamiltonian (as a representation of Supplementary Equation 15 in the rotating frame)

$$\mathcal{V}_{ij,kl} = V^{\text{dd}}_{ij,kl} - (\Delta_i + \Delta_j)\delta_{ik}\delta_{jl} \tag{30}$$

whose eigenvalues are the potential energy curves shown e.g. in Fig. 1c of the main text. It differs from the raw dipole potential essentially in that the pair energies approach the noninteracting pair energies for $r \to \infty$ rather than dropping to zero. In the limit of weak fields, i.e. when the Rydberg exciton density per blockade volume is small, the leading-order nonlinear polarization is determined by the following set of adiabatic equations of motion

$$\begin{aligned}\frac{d}{dt}\left(\hat{X}^\dagger_{i'}(\mathbf{r}')\hat{X}_i(\mathbf{r}')\hat{X}_j(\mathbf{r})\right) =& -\Omega_{i'}\frac{-2g\mathcal{E}^*(\mathbf{r}')\hat{X}_i(\mathbf{r}')\hat{X}_j(\mathbf{r}) + \sum_p \Omega_p \hat{X}^\dagger_p(\mathbf{r}')\hat{X}_i(\mathbf{r}')\hat{X}_j(\mathbf{r})}{2\Gamma^*}\\ &-\Omega_i\frac{-2g\mathcal{E}(\mathbf{r}')\hat{X}^\dagger_{i'}(\mathbf{r}')\hat{X}_j(\mathbf{r}) + \sum_p \Omega_p \hat{X}^\dagger_{i'}(\mathbf{r}')\hat{X}_p(\mathbf{r}')\hat{X}_j(\mathbf{r})}{2\Gamma}\\ &-\Omega_j\frac{-2g\mathcal{E}(\mathbf{r})\hat{X}^\dagger_{i'}(\mathbf{r}')\hat{X}_i(\mathbf{r}') + \sum_p \Omega_p \hat{X}^\dagger_{i'}(\mathbf{r}')\hat{X}_i(\mathbf{r}')\hat{X}_p(\mathbf{r})}{2\Gamma}\\ &-\left(\frac{\Gamma^*_{i'}}{2} + \frac{\bar{\gamma}_i}{2} + \frac{\bar{\gamma}_j}{2}\right)\hat{X}^\dagger_{i'}(\mathbf{r}')\hat{X}_i(\mathbf{r}')\hat{X}_j(\mathbf{r}) - i\sum_{pp'}\mathcal{V}_{ji,pp'}(|\mathbf{r}-\mathbf{r}'|)\hat{X}^\dagger_{i'}(\mathbf{r}')\hat{X}_{p'}(\mathbf{r}')\hat{X}_p(\mathbf{r})\end{aligned} \tag{31}$$

$$\begin{aligned}\frac{d}{dt}\left(\hat{X}_i(\mathbf{r}')\hat{X}_j(\mathbf{r})\right) =& -\Omega_i\frac{-2g\mathcal{E}(\mathbf{r}')\hat{X}_j(\mathbf{r}) + \sum_p \Omega_p \hat{X}_p(\mathbf{r}')\hat{X}_j(\mathbf{r})}{2\Gamma} - \Omega_j\frac{-2g\mathcal{E}(\mathbf{r})\hat{X}_i(\mathbf{r}') + \sum_p \Omega_p \hat{X}_i(\mathbf{r}')\hat{X}_p(\mathbf{r})}{2\Gamma}\\ &-\left(\frac{\bar{\gamma}_i}{2} + \frac{\bar{\gamma}_j}{2}\right)\hat{X}_i(\mathbf{r}')\hat{X}_j(\mathbf{r}) - i\sum_{pp'}\mathcal{V}_{ji,pp'}(|\mathbf{r}-\mathbf{r}'|)\hat{X}_{p'}(\mathbf{r}')\hat{X}_p(\mathbf{r})\end{aligned} \tag{32}$$

$$\begin{aligned}\frac{d}{dt}\left(\hat{X}^\dagger_i(\mathbf{r}')\hat{X}_j(\mathbf{r})\right) =& -\Omega_i\frac{-2g\mathcal{E}^*(\mathbf{r}')\hat{X}_j(\mathbf{r}) + \sum_p \Omega_p \hat{X}^\dagger_p(\mathbf{r}')\hat{X}_j(\mathbf{r})}{2\Gamma^*} - \Omega_j\frac{-2g\mathcal{E}(\mathbf{r})\hat{X}_i(\mathbf{r}') + \sum_p \Omega_p \hat{X}^\dagger_i(\mathbf{r}')\hat{X}_p(\mathbf{r})}{2\Gamma}\\ &-\left(\frac{\Gamma^*_i}{2} + \frac{\Gamma_j}{2}\right)\hat{X}_i(\mathbf{r}')\hat{X}_j(\mathbf{r})\end{aligned} \tag{33}$$

$$\begin{aligned}\frac{d}{dt}\left(\hat{X}^\dagger_i(\mathbf{r})\hat{X}_j(\mathbf{r})\right) =& -\Omega_i\frac{-2g\mathcal{E}^*(\mathbf{r})\hat{X}_j(\mathbf{r}) + \sum_p \Omega_p \hat{X}^\dagger_p(\mathbf{r})\hat{X}_j(\mathbf{r})}{2\Gamma^*} - \Omega_j\frac{-2g\mathcal{E}(\mathbf{r})\hat{X}^\dagger_i(\mathbf{r}) + \sum_p \Omega_p \hat{X}^\dagger_i(\mathbf{r})\hat{X}_p(\mathbf{r})}{2\Gamma}\\ &-\left(\frac{\Gamma^*_i}{2} + \frac{\Gamma_j}{2}\right)\hat{X}_i(\mathbf{r})\hat{X}_j(\mathbf{r})\end{aligned} \tag{34}$$

After taking expectation values and considering the excitonic adiabatic steady state, we are left with a closed set of algebraic equations. The key step is to transform the steady state equations from the product basis to the pair basis (Supplementary Equation 19), as for example

$$\sum_{pp'} \mathcal{V}_{ji,pp'}(|\mathbf{r}-\mathbf{r}'|)\langle \hat{X}^\dagger_{i'}(\mathbf{r}')\hat{X}_{p'}(\mathbf{r}')\hat{X}_p(\mathbf{r})\rangle = \sum_\mu U_\mu(|\mathbf{r}-\mathbf{r}'|)c_{ji,\mu}Y_{gi',\mu}(\mathbf{r},\mathbf{r}') \tag{35}$$

where $Y_{gi',\mu}(\mathbf{r},\mathbf{r}')$ is the expectation value of the operator destroying a pair state μ shared between excitons in $\mathbf{r}$ and $\mathbf{r}'$ and replacing it by a product state of a ground state and a Rydberg state i' in the same positions. The insights gained from the new basis are twofold: Firstly, the equations become diagonal (the residual off-diagonal couplings are very weak) and can, thus, be solved analytically. Secondly, the pair basis provides the correct energy scale to consider. Rather than using the unfeasible number of all states we restrict our attention to states within an energy window around the two-photon resonance. We solve the system of equations in terms the single-particle correlators which we evaluate using the ansatz of the (approximate and non-interacting) EIT ground state, such that we are able to formulate a nonlinear equation for the medium's polarization $\mathcal{P}(\mathbf{r}) = \langle \hat{X}_g(\mathbf{r})\rangle$ (Equation 1 in main text)

$$\mathcal{P}(\mathbf{r}) = \chi^{(1)}\mathcal{E}(\mathbf{r}) + \int d\mathbf{r}'\chi^{(3)}(|\mathbf{r}-\mathbf{r}'|)|\mathcal{E}(\mathbf{r}')|^2\mathcal{E}(\mathbf{r}) \tag{36}$$

For the linear susceptibility $\chi^{(1)}$ we obtain

$$\chi^{(1)} = -2g\frac{i}{\Gamma}\left[1-\sum_k \frac{\Omega_k^2}{\Omega_k^2+\Gamma_k\Gamma}F_k^{(1)}\right] \qquad F_k^{(1)} = 1-\sum_{k'\neq k}\frac{\Omega_{k'}^2}{\Omega_{k'}^2+\Gamma_{k'}\Gamma}, \tag{37}$$

while the nonlinear susceptibility $\chi^{(3)}$ is given by

$$\chi^{(3)}(|\mathbf{r}-\mathbf{r}'|) = -16g^3\sum_{k,l}\frac{\Omega_k F_k^{(3)}}{\Omega_k^2+\Gamma_k\Gamma}\sum_\mu \left[U_\mu(|\mathbf{r}-\mathbf{r}'|)+(\Delta_k+\Delta_l)\right]c^*_{[kl],\mu}Y_{gl,\mu}(|\mathbf{r}-\mathbf{r}'|) \tag{38}$$

$$Y_{gl,\mu}(|\mathbf{r}-\mathbf{r}'|) = -\frac{\Omega_l}{\Omega_l^2+\Gamma_l^*\Gamma^*}\cdot\frac{\sum_{k'l'}c_{[k'l'],\mu}\cdot A_{k',l'}}{\tilde{\Omega}^2(\mu)+\Gamma\left[iU_\mu(|\mathbf{r}-\mathbf{r}'|)+\sum_{k'l'}|c_{[k'l'],\mu}|^2\gamma_{k'}\right]} \tag{39}$$

where we defined

$$F_k^{(3)} = 1-\Omega_k\sum_{k'\neq k}\frac{\Omega_{k'}}{\Omega_{k'}^2+\Gamma_{k'}\Gamma} \tag{40}$$

$$A_{k',l'} = \frac{\Omega_{k'}\Omega_{l'}}{2}\cdot\frac{[\Omega_{k'}^2+\Gamma_{k'}\Gamma]+[\Omega_{l'}^2+\Gamma_{l'}\Gamma]}{[\Omega_{k'}^2+\Gamma_{k'}\Gamma]\cdot[\Omega_{l'}^2+\Gamma_{l'}\Gamma]} \tag{41}$$

$$\tilde{\Omega}^2(\mu) = \sum_{k,l,m}c_{[kl],\mu}c^*_{[km],\mu}\Omega_l\Omega_m. \tag{42}$$

Both single-particle and two-particle terms have very weak off-diagonal coupling terms, which we capture perturbatively with the help of $F_k^{(1)}$ and $F_k^{(3)}$, both of which are typically quite small. It turns out that there are only odd terms affecting the light field. In fact, all even orders vanish as can be shown from the inversion symmetry of a medium. While one could object that TMDC materials break inversion symmetry leading to important features including second harmonic generation [16], our results are restricted to the limit of Rydberg excitons, which span hundreds of thousands of crystal cells, and are, thus, very well approximated by (inversion-symmetric) continuous wavefunctions.

To arrive at the desired effective equation for the light field, we must consider that a cavity photon is converted into an exciton and, thus, evolves much more slowly. This is very closely related to the slow-light effect known from EIT propagation experiments [17] and, mathematically, it is found by solving all the coupled equations to first order in the slowly varying cavity field. Neglecting interactions in Supplementary Equation 29 we first iteratively solve the coherence for a single potential surface

$$\tilde{X}_k^{(1),\text{ single potential}}(r) = \frac{2\Omega_k}{\Omega_k^2+\Gamma\Gamma_k}g\mathcal{E}(\mathbf{r}) \tag{43}$$

and then iteratively solve in the non-diagonal terms as done above giving

$$\tilde{X}_k^{(1)}(\mathbf{r}) = X_k^{(1)}(\mathbf{r}) - \frac{4\Gamma\Omega_k}{(\Omega_k^2+\Gamma\Gamma_k)^2} \underbrace{\left[1-(\Omega_k^2+\Gamma\Gamma_k)\sum_{k'\neq k}\frac{\Omega_{k'}^2}{(\Omega_{k'}^2+\Gamma\Gamma_{k'})^2}\right]}_{\equiv F_k^3} g\partial_t\mathcal{E}(\mathbf{r}). \tag{44}$$

The remaining terms do not contribute to any linear terms in $\partial_t\mathcal{E}$. As done above, we substitute into Supplementary Equation 27 and find as the only modification

$$\tilde{X}_g^{(1)} = X_g^{(1)} - 4i\sum_k \frac{\Omega_k^2}{(\Omega_k^2+\Gamma\Gamma_k)^2}\cdot F_k^3\cdot g\partial_t\mathcal{E}. \tag{45}$$

We define the (slow-light) factor $\nu = 1+4g^2\sum_k \frac{\Omega_k^2}{(\Omega_k^2+\Gamma\Gamma_k)^2}\cdot F_k^3$, such that the in-cavity dynamics is given by

$$\nu\partial_t\mathcal{E}(\mathbf{r}) = i\frac{\hbar}{2m_{\text{ph}}}\nabla^2\mathcal{E}(\mathbf{r}) - \frac{\Gamma_{\text{cav}}}{2}\mathcal{E}(\mathbf{r}) + \eta E_0^{\text{inc}} - ig\left(\chi^{(1)} + \int d^2r'\chi^{(3)}(|\mathbf{r}-\mathbf{r}'|)|\mathcal{E}(\mathbf{r}')|^2\right)\mathcal{E}(\mathbf{r}). \tag{46}$$

We define the nonlinear interaction $W(r) = \Re\left(\frac{g}{\nu}\chi^{(3)}(r)\right)$, which is shown in Fig. 1d (main text) and is the basis for Fig. 2 (main text). The corresponding imaginary component constitutes a nonlinear absorption term $\Gamma_{\text{nl}}(r) = \Im\left(\frac{g}{\nu}\chi^{(3)}(r)\right)$. The inset to Fig. 2a (main text) is calculated by solving Supplementary Equation 46 for the (flat) cavity steady state $\partial_t\mathcal{E}(r) = 0$. The transmission is proportional to the ratio of the cavity steady state density and the input driving strength

$$\begin{aligned} T^{-1} \propto \frac{(\eta E_0^{\text{inc}})}{|\mathcal{E}_0|^2} &= g^2\left|\int d\mathbf{r}\chi^{(3)}(|\mathbf{r}|)\right|^2|\mathcal{E}_0|^4 + \left(-\frac{\kappa}{2}+g\chi_{\text{I}}^{(1)}\right)^2 + \left(g\chi_{\text{R}}^{(1)}-\Delta_{\text{cav}}\right)^2 \\ &+ 2g\left[\int d\mathbf{r}\chi_{\text{R}}^{(3)}(|\mathbf{r}|)\cdot\left(g\chi_{\text{R}}^{(1)}-\Delta_{\text{cav}}\right) + \int d\mathbf{r}\chi_{\text{I}}^{(3)}(|\mathbf{r}|)\cdot\left(-\frac{\kappa}{2}+g\chi_{\text{I}}^{(1)}\right)\right]|\mathcal{E}_0|^2 \end{aligned} \tag{47}$$

and is evaluated as a function of the cavity frequency (via Δ_{cav}) in the inset to Fig. 2a of the main text. The point of maxial transmission is therefore given by

$$\Delta_{\text{cav}} = g\chi_{\text{R}}^{(1)} + g\int d\mathbf{r}\chi_{\text{R}}^{(3)}(|\mathbf{r}|)|\mathcal{E}_0|^2 \tag{48}$$

For typical parameters the slow light factor can be approximated by the real number $\nu\approx 4\frac{g^2}{\Omega^2}$, such that the nonlinear cavity shift can be expressed in terms of $\alpha = \int d^2rW(r)$ as

$$\delta_{\text{nl}} \approx \frac{4\alpha g^2}{\Omega^2}|\mathcal{E}_0|^2 \tag{49}$$

as given in the main text.

Supplementary Note 5: Some Limiting Cases

To provide more insights of the complicated expressions of the nonlinear potential in Supplementary Equation 38, we analyze some of its features: At large distances the photonic potential goes to zero because the dipole interaction vanishes. Mathematically, we can see this from Supplementary Equations (38-39), where $c^*_{[kl],\mu}\to\delta_{[kl],\mu}$ and $U_\mu\to -(\Delta_k+\Delta_l)$, leaving no total contribution. Saturation as a single-particle effect cannot enter in the assumed limit of weak driving fields. The other limit is the plateau at small distances, where the dipole interaction pushes the molecular states far away from their product values. At even smaller distances, the states are mixed even more strongly, some re-enter the two-photon resonance. First, we consider the case $V^{\text{dd}} > \bar{\gamma}$, i.e. the interaction shift is larger than the decay-broadened Rydberg line. From the general equation we observe that for relatively small Rydberg decay and away from new resonances the terms $\Gamma_k\Gamma$ suppress contributions other than the resonant one. The nonlinear susceptibility thus reads

$$\chi^{(3)}(r) = \frac{8g^3\Omega^4}{(\Omega^2+\Gamma\bar{\gamma})\cdot|\Omega^2+\Gamma\bar{\gamma}|^2}\cdot\frac{2U(r)}{\Omega^2+\bar{\gamma}\Gamma+i\Gamma U(r)}, \tag{50}$$

where we named the only active Rabi coupling to the Rydberg potential Ω. The nonlinear optical response, characterized by $W(r)$ and $\Gamma_{\mathrm{nl}}(r)$, is

$$W(r) = \frac{4g^2\Omega^2 U(r)\cdot\left[\bar{\gamma}^2(\gamma^2+4\Delta^2)+2(\gamma\bar{\gamma}+\Delta U(r))\Omega^2+\Omega^4\right]}{|\Omega^2+\Gamma\bar{\gamma}|^2\cdot|\Omega^2+\bar{\gamma}\Gamma+i\Gamma U(r)|^2} \tag{51}$$

$$\Gamma_{\mathrm{nl}}(r) = -\frac{4g^2\Omega^2 U^2(r)\cdot\left[\gamma^2\bar{\gamma}+4\bar{\gamma}\Delta^2+\gamma\Omega^2\right]}{|\Omega^2+\Gamma\bar{\gamma}|^2\cdot|\Omega^2+\bar{\gamma}\Gamma+i\Gamma U(r)|^2}, \tag{52}$$

where we used the very accurate approximation $\nu = \frac{4g^2\Omega^2}{(\Omega^2+\Gamma\bar{\gamma})^2}$ for simplicity. First consider the plateau height in the case of small separations, i.e. $U\to\infty$

$$W(0) = \frac{8g^2\Delta\Omega^4}{|\Omega^2+\Gamma\bar{\gamma}|^2\cdot|\Gamma|^2} \tag{53}$$

$$\Gamma_{\mathrm{nl}}(0) = -\frac{4g^2\Omega^2\left[\gamma^2\bar{\gamma}+4\bar{\gamma}\Delta^2+\gamma\Omega^2\right]}{|\Omega^2+\Gamma\bar{\gamma}|^2\cdot|\Gamma|^2} \tag{54}$$

Next, we compare our multilevel theory with the simplified version of a single van der Waals potential $U = C_6/R^6$

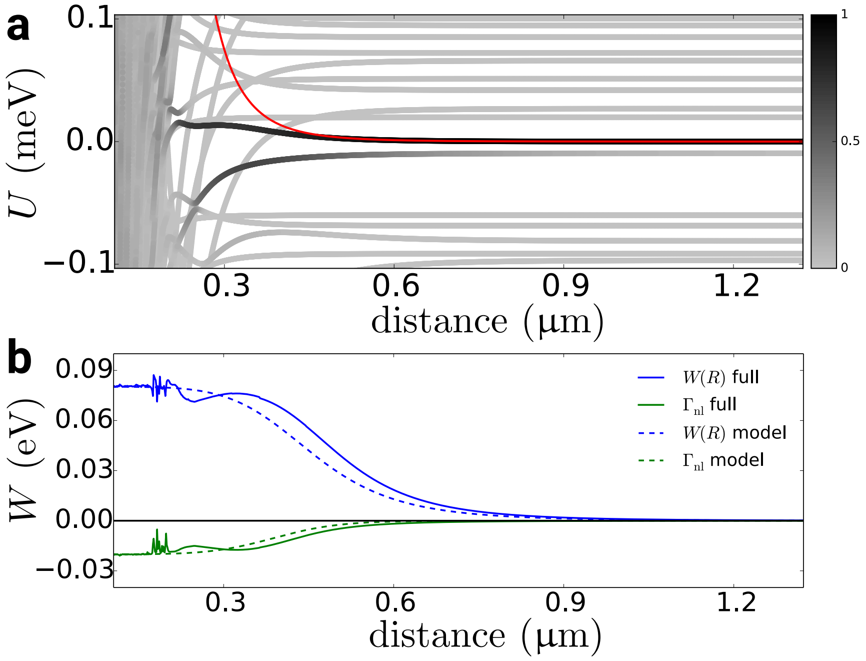

Supplementary Figure 2: Comparison of the multiple-line theory with a single-line model. (a) The excitonic potential at K^+, $m=1$ and $n=10$ can be approximated at large distance by a van der Waals curve $U\approx 4.96\cdot 10^{-5}$ meV$(\mu\mathrm{m})^6/r^6$ (red). The gray coloring indicates the relative coupling from stongly- (black) to non-coupling curves (light gray). (b) For excitonic ground state decay rate $\gamma = 300$ GHz, Rydberg decay $\bar{\gamma} = 0.3$ GHz, single-photon detuning $\Delta = 10\gamma$, coupling Rabi frequency $\Omega = 10\delta_{\mathrm{EIT}}$ the optical nonlinearity is given by the solid curves. The real part (blue) defines the potential, while the imaginary part describes nonlinear loss (green). The single-line model (dashed lines) agrees well at large and short distances but deviates in the intermediate region.

(Supplementary Figure 2 a) by fitting C_6 at great distances and continuing the potential into smaller separations. At large distances the optical potentials of both cases tends to zero, at small distances both enter a plateau region whose height is entirely determined by the laser parameters. Remarkably, the plateau height is precisely the same for the multilevel and the non-mixing single potential calculation (Supplementary Figure 2 b). This is because the physical mechanism at the heart of the plateau is the loss of a coupling pair state, an effect which can either be brought about

by a shift (van der Waals fit) or the combined action of shifting and state mixing (full potential). Since the plateau height is identical we use the simpler single potential relations to analyze its scaling relations.

For the special case $\bar{\gamma} = 0$ we find the familiar scaling relations in the dispersive regime $\Delta \gg \gamma$

$$W(0) \approx \frac{2g^2}{\Delta} \qquad \Gamma_{\text{nl}}(0) \approx -\frac{g^2\gamma}{\Delta^2}. \tag{55}$$

In this limit, we can get a dominant real part by increasing the detuning. If $\bar{\gamma} \neq 0$, however, the plateau height scaling takes a different form and we can derive from Supplementary Equations (53-54) that neglecting the Rydberg decay is legitimate if $\bar{\gamma} < \Omega^2/|\Gamma| = \delta_{\text{EIT}}$ and $\Delta > \gamma$ (dispersive regime).

Another characteristic figure is the potential height $U^{\text{c}} = U(R_{\text{c}})$ needed to form the potential, which we define via $W(R_{\text{c}}) = \frac{1}{2}W(0)$. This shift determines the potential's characteristic length scale R_{c}. Even for the single potential surface this is a rather lengthy expression, we examine here the special case $\bar{\gamma} = 0$ finding

$$U^{\text{c}} = \frac{\Omega^2}{2\Delta} \cdot \frac{\pm\sqrt{1 + \left(\frac{\gamma}{2\Delta}\right)^2 + \left(\frac{\gamma}{2\Delta}\right)^4} - \left(\frac{\gamma}{2\Delta}\right)^2}{1 + \left(\frac{\gamma}{2\Delta}\right)^2} \to \pm\frac{\Omega^2}{2\Delta}. \tag{56}$$

In the dispersive regime we thus recover the simple physics outlined in the main text: The interactions must exceed leave the EIT window $\delta_{\text{EIT}} = \Omega^2/\Delta$ for a strong optical response. Although the full potential rises less steeply than the van der Waals fit we observe that its optical plateau typically rises at greater distances. This is, again, due to the loss of coupling strength thanks to state mixing. Some more detailed potential features, such as local minima, are also not captured by the single potential model. We conclude that while only the full response accounting for state mixing and resonances can capture all features, the above estimates based on the van der Waals model provide a reliable approximation for most important aspects.

Supplementary Note 6: Dipole vs. Exchange Interactions

We evaluated the optical theory outlined above for the dipole-dipole interactions derived in Supplementary Note 2. This approach to calculate interactions between excited exciton states differs substantially from what is otherwise used for ground state excitons, where exchange effects dominate. One can characterize the importance of exchange effects by the wave function overlap

$$\mathcal{O} = \int \mathrm{d}\mathbf{x} |\psi(\mathbf{x})| \psi(\mathbf{x}+\mathbf{r})|^2 \tag{57}$$

between two excitons whose center of mass is separated by a distance $|\mathbf{r}|$. For comparison we can also consider the two-exciton correlation function

$$g^{(2)}_{\text{X}} = \langle \hat{X}^\dagger(\mathbf{x})\hat{X}^\dagger(\mathbf{x}+\mathbf{r})\hat{X}(\mathbf{x}+\mathbf{r})\hat{X}(\mathbf{x})\rangle \tag{58}$$

that gives the relative probability to find two excitons at a distance $|\mathbf{r}|$. As demonstrated in Supplementary Figure 3, the wave function overlap (and thereby exchange effects) vanishes well inside the blockade radius where two excitons cannot be excited. This arguments holds for a wide range of principal quantum numbers since both characteristic length scales exhibit an approximate n-scaling of $\sim n^2$, as illustrated exemplarily by the left and right panel of the figure. In addition we indicate in the upper row the LeRoy radius [18]

$$r_{\text{LR}} = 4\int \mathrm{d}\mathbf{x}|\mathbf{x}|^2|\psi(\mathbf{x})|^2 \tag{59}$$

which also shows that exchange effects are negligible outside the blockade radius.

Supplementary Note 7: Photon Correlations

In this Supplementary Note, we evaluate the quantum photon statistics of the cavity system described by Supplementary Equations (28-29). We exploit that there is a natural separation of times scales. For realistic cavities (bad cavity limit) the light field will reach a steady state fast, usually on timescale given by κ^{-1}. This is followed by a

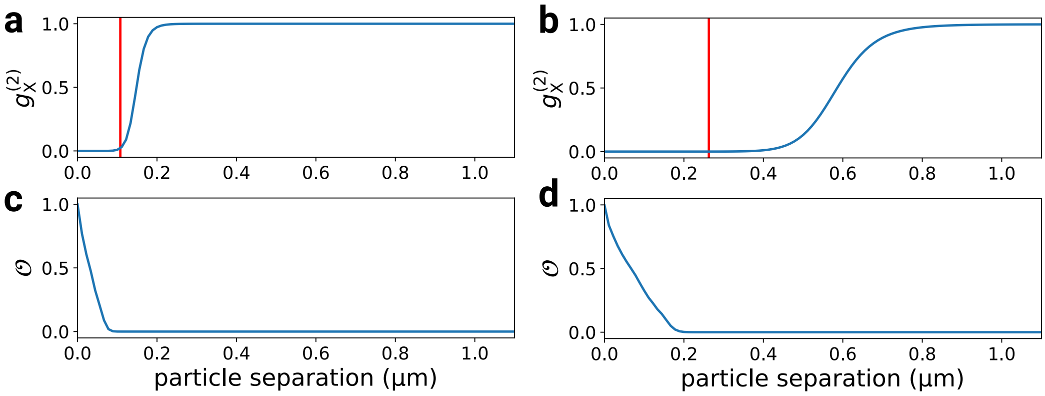

Supplementary Figure 3: Exchange effects are blocked by Rydberg interactions. (a) The probability of exciting two excitons, $g_{\mathrm{X}}^{(2)}$, is strongly suppressed at exciton sepations shorter than the LeRoy radius (red) for the addressed state $n = 6$, $m = 1$. The optical parameters are chosen as in Fig. 1 of the paper: $\Omega/(2\pi) = 20$ GHz, $\Delta/(2\pi) = 700$ GHz, $\gamma/(2\pi) = 300$ GHz and $\bar{\gamma}/(2\pi) = 1.4$ GHz. Outside this regime, there is no significant overlap of the wavefunctions (c). For higher addressed quantum numbers (here $n = 10$ and $\bar{\gamma}/(2\pi) = 0.3$ GHz) but under otherwise equal conditions, the blockade radius quickly grows (b). The difference between the blockade radius and LeRoy radius demarcating the separations dominated by exchange effects increases (d).

relaxation of the excitons' internal dynamics on a much longer timescale and finally the onset of transverse density currents in the light intensity driven by the kinetic energy operator on a yet longer timescale [19]. Here, we focus on times shorter than necessary for the formation of intensity modulation, such that we can neglect the photonic kinetic energy term. The formal solution of Supplementary Equation 28 reads, for coherent driving

$$\hat{\mathcal{E}}(\mathbf{r},t) = e^{-\frac{\Gamma_{\mathrm{cav}}\Gamma+4g^2}{2\Gamma}t}\hat{\mathcal{E}}(\mathbf{r},0) + \int_0^t e^{-\frac{\Gamma_{\mathrm{cav}}\Gamma+4g^2}{2\Gamma}(t-t')}\left(\frac{g}{\Gamma}\sum_i \Omega_i \hat{X}_i(\mathbf{r},t') + \eta E^{\mathrm{in}}(\mathbf{r})\right)dt'. \tag{60}$$

Since the cavity is initially empty we drop the term proportional to $\hat{\mathcal{E}}(r,0)$. Because of the timescale separation it now is possible to solve the integral in the Markov approximation. We first formulate the integral with a memory kernel (which does not change the limits of integration), $\tau = t - t'$

$$\int_0^t e^{-\frac{\Gamma_{\mathrm{cav}}\Gamma+4g^2}{2\Gamma}\tau}\left(\frac{g}{\Gamma}\sum_i \Omega_i \hat{X}_i(\mathbf{r},t-\tau) + \eta E^{\mathrm{in}}(\mathbf{r})\right)d\tau \tag{61}$$

and replace $\hat{X}_i(\mathbf{r},t-\tau) \to \hat{X}_i(\mathbf{r},t)$ under the integral. Carrying out the integral, we find the solution after the very short initial time, i.e. for $t \gg \kappa^{-1}$

$$\hat{\mathcal{E}}(\mathbf{r},t) = \frac{2g}{\Gamma_{\mathrm{cav}}\Gamma + 4g^2}\sum_i \Omega_i \hat{X}_i(\mathbf{r},t) + \frac{2\Gamma\eta}{\Gamma_{\mathrm{cav}}\Gamma + 4g^2}E^{\mathrm{in}}(\mathbf{r}). \tag{62}$$

Plugging this adiabtic solution into Supplementary Equation 29, we are left with an equation of motion only in the excitonic degrees of freedom

$$\begin{aligned}\partial_t \hat{X}_k(\mathbf{r}) = &\frac{\Omega_k}{\Gamma}\left(-\frac{1}{2} + \frac{2g^2}{\Gamma_{\mathrm{cav}}\Gamma + 4g^2}\right)\sum_i \Omega_i \hat{X}_i(\mathbf{r}) - \frac{\Gamma_k}{2}\hat{X}_k(\mathbf{r}) + \frac{2\eta g\Omega_k}{\Gamma_{\mathrm{cav}}\Gamma + 4g^2}E^{\mathrm{in}}(\mathbf{r}) \\ &- i\sum_{i\le j,i'}\int d^2r' \hat{X}_{i'}^\dagger(\mathbf{r}')\frac{V^{\mathrm{dd}}_{i'k,ij}(|\mathbf{r}'-\mathbf{r}|)}{\hbar}\hat{X}_i(\mathbf{r}')\hat{X}_j(\mathbf{r}).\end{aligned} \tag{63}$$

We note that Supplementary Equation 63 is formally equivalent to Supplementary Equation 29, although the former includes quantum correlations between excitons and the cavity field. Thanks to this formal identity we can employ the same solution strategy as above, replacing only certain variables. As demonstrated in Supplementary Note 5,

reduction to a system with one primary Rydberg level (created by $\hat{X}_s^\dagger$) interacting via van der Waals interactions $V(R) = \hbar\omega_0/(R/R_c)^6$ produces a good approximation to the full solution. For simplicity of notation and computation we will consider this simplified model in the following

$$\hat{\mathcal{E}}(\mathbf{r}) = \underbrace{\frac{2g\Omega}{\Gamma_{\mathrm{cav}}\Gamma + 4g^2}}_{\equiv\gamma} \hat{X}_s(\mathbf{r}) + \underbrace{\frac{2\Gamma\eta}{\Gamma_{\mathrm{cav}}\Gamma + 4g^2}}_{\equiv\delta} E^{\mathrm{in}}(\mathbf{r}) \tag{64}$$

$$\partial_t \hat{X}_s(\mathbf{r}) = \underbrace{\left(\frac{\Omega^2}{\Gamma}\left(-\frac{1}{2} + \frac{2g^2}{\Gamma_{\mathrm{cav}}\Gamma + 4g^2}\right) - \frac{\Gamma_s}{2}\right)}_{\equiv\alpha} \hat{X}_s(\mathbf{r}) + \underbrace{\frac{2\eta g\Omega}{\Gamma_{\mathrm{cav}}\Gamma + 4g^2}}_{\equiv\beta} E^{\mathrm{in}}(\mathbf{r}) - i\int d\mathbf{r}' \hat{X}_s^\dagger(\mathbf{r}') \frac{\omega_0 R_{\mathrm{c}}^6}{|\mathbf{r}-\mathbf{r}'|^6} \hat{X}_s(\mathbf{r}')\hat{X}_s(\mathbf{r}). \tag{65}$$

The photon statistics $g^{(2)}(0)$ requires spatially resolved information on the photonic operators, in particular on the correlation function

$$\left\langle \hat{\mathcal{E}}^\dagger(\mathbf{r}_1)\hat{\mathcal{E}}^\dagger(\mathbf{r}_2)\hat{\mathcal{E}}(\mathbf{r}_1)\hat{\mathcal{E}}(\mathbf{r}_2) \right\rangle, \tag{66}$$

which can be expressed in terms of excitonic operators by virtue of Supplementary Equation 64

$$\begin{aligned} &|\gamma|^4 \hat{X}_s^\dagger(\mathbf{r}_1)\hat{X}_s^\dagger(\mathbf{r}_2)\hat{X}_s(\mathbf{r}_1)\hat{X}_s(\mathbf{r}_2) + |\gamma|^2\gamma^*\delta E^{\mathrm{in}}(\mathbf{r}_2)\hat{X}_s^\dagger(\mathbf{r}_1)\hat{X}_s^\dagger(\mathbf{r}_2)\hat{X}_s(\mathbf{r}_1) \\ &+|\gamma|^2\delta^*\gamma E^{\mathrm{in}*}(\mathbf{r}_2)\hat{X}_s^\dagger(\mathbf{r}_1)\hat{X}_s(\mathbf{r}_1)\hat{X}_s(\mathbf{r}_2) + |\gamma|^2|\delta|^2 E^{\mathrm{in}}(\mathbf{r}_2)E^{\mathrm{in}*}(\mathbf{r}_2)\hat{X}_s^\dagger(\mathbf{r}_1)\hat{X}_s(\mathbf{r}_1) + ... \end{aligned} \tag{67}$$

Out of the 16 terms in this expansion all but the very first term have already been evaluated in Supplementary Note 4. This first term describes the excitonic blockade or the expectation value of finding Rydberg-excited excitons in positions $\mathbf{r}_1$ and $\mathbf{r}_2$ and is, in fact, a linear combination of the other (known) two-particle terms. For completeness, all necessary terms are given below

$$\left\langle \hat{X}_s^\dagger(\mathbf{r}_1)\hat{X}_s(\mathbf{r}_1) \right\rangle = \frac{-\beta E^{\mathrm{in}}(\mathbf{r}_1)\left\langle \hat{X}_s^\dagger(\mathbf{r}_1)\right\rangle - \beta^* E^{\mathrm{in}*}(\mathbf{r}_1)\left\langle \hat{X}_s(\mathbf{r}_1)\right\rangle}{\alpha+\alpha^*} \tag{68}$$

$$\left\langle \hat{X}_s(\mathbf{r}_1)\hat{X}_s(\mathbf{r}_2) \right\rangle = \frac{\beta\left(E^{\mathrm{in}}(\mathbf{r}_1)\left\langle \hat{X}_s^\dagger(\mathbf{r}_2)\right\rangle + E^{\mathrm{in}}(\mathbf{r}_2)\left\langle \hat{X}_s(\mathbf{r}_1)\right\rangle\right)}{-2\alpha + iV(|\mathbf{r}_1-\mathbf{r}_2|)} \tag{69}$$

$$\left\langle \hat{X}_s^\dagger(\mathbf{r}_2)\hat{X}_s(\mathbf{r}_1) \right\rangle = \frac{-\beta E^{\mathrm{in}}(\mathbf{r}_1)\left\langle \hat{X}_s^\dagger(\mathbf{r}_2)\right\rangle - \beta^* E^{\mathrm{in}*}(\mathbf{r}_2)\left\langle \hat{X}_s(\mathbf{r}_1)\right\rangle}{\alpha+\alpha^*} \tag{70}$$

$$\begin{aligned} \left\langle \hat{X}_s^\dagger(\mathbf{r}_2)\hat{X}_s(\mathbf{r}_1)\hat{X}_s(\mathbf{r}_2) \right\rangle = \frac{1}{-2\alpha-\alpha^*+iV(|\mathbf{r}_1-\mathbf{r}_2|)} &\Big[\beta E^{\mathrm{in}}(\mathbf{r}_1)\left\langle \hat{X}_s^\dagger(\mathbf{r}_2)\hat{X}_s(\mathbf{r}_2)\right\rangle \\ &+\beta^* E^{\mathrm{in}*}(\mathbf{r}_2)\left\langle \hat{X}_s(\mathbf{r}_1)\hat{X}_s(\mathbf{r}_2)\right\rangle \\ &+\beta E^{\mathrm{in}}(\mathbf{r}_2)\left\langle \hat{X}_s^\dagger(\mathbf{r}_2)\hat{X}_s(\mathbf{r}_1)\right\rangle\Big] \end{aligned} \tag{71}$$

$$\begin{aligned} \left\langle \hat{X}_s^\dagger(\mathbf{r}_1)\hat{X}_s^\dagger(\mathbf{r}_2)\hat{X}_s(\mathbf{r}_1)\hat{X}_s(\mathbf{r}_2) \right\rangle = \frac{1}{2(\alpha+\alpha^*)} &\Big[-\beta^* E^{\mathrm{in}*}(\mathbf{r}_2)\left\langle \hat{X}_s^\dagger(\mathbf{r}_2)\hat{X}_s(\mathbf{r}_1)\hat{X}_s(\mathbf{r}_2)\right\rangle \\ &-\beta E^{\mathrm{in}}(\mathbf{r}_1)\left\langle \hat{X}_s^\dagger(\mathbf{r}_1)\hat{X}_s^\dagger(\mathbf{r}_2)\hat{X}_s(\mathbf{r}_2)\right\rangle \\ &-\beta^* E^{\mathrm{in}*}(\mathbf{r}_1)\left\langle \hat{X}_s^\dagger(\mathbf{r}_1)\hat{X}_s(\mathbf{r}_1)\hat{X}_s(\mathbf{r}_2)\right\rangle \\ &-\beta E^{\mathrm{in}}(r_2)\left\langle \hat{X}_s^\dagger(\mathbf{r}_1)\hat{X}_s^\dagger(\mathbf{r}_2)\hat{X}_s(\mathbf{r}_1)\right\rangle\Big]. \end{aligned} \tag{72}$$

This system of equations leads to a nonlinear equation in the single-particle correlator. We solve this equation for weak driving fields by plugging in the (approximate) noninteracting solution. It turns out that the external driving

field can be factored out of all terms and cancels with the denominator

$$h^{(2)}(\mathbf{r}_1,\mathbf{r}_2) \equiv \frac{\left\langle \hat{\mathcal{E}}^\dagger(\mathbf{r}_1)\hat{\mathcal{E}}^\dagger(\mathbf{r}_2)\hat{\mathcal{E}}(\mathbf{r}_1)\hat{\mathcal{E}}(\mathbf{r}_2)\right\rangle}{\left\langle \hat{\mathcal{E}}^\dagger(\mathbf{r}_1)\hat{\mathcal{E}}(\mathbf{r}_1)\right\rangle \left\langle \hat{\mathcal{E}}^\dagger(\mathbf{r}_2)\hat{\mathcal{E}}(\mathbf{r}_2)\right\rangle}$$
$$= \frac{1}{|-\gamma\frac{\beta}{\alpha}+\delta|^4}\left(4\left|\frac{\beta\gamma\delta}{\alpha}\right|^2 + |\delta|^4\right. \tag{73}$$
$$+4\Re\left[\left(\frac{\beta^*|\beta/\alpha|^2}{-2\alpha^*-\alpha-iV(|\mathbf{r}_1-\mathbf{r}_2|)} + \frac{\beta^{*2}/\alpha^*}{2\alpha^*+iV(|\mathbf{r}_1-\mathbf{r}_2|)}\right)\cdot\left(-\frac{\beta|\gamma|^4}{\alpha+\alpha^*}+2|\gamma|^2\gamma^*\delta\right)\right.$$
$$\left.\left.(\gamma^*\delta)^2\frac{\beta^{*2}/\alpha^*}{2\alpha^*+iV(r_1-r_2)} - \frac{\gamma^*\delta|\delta|^2\beta^*}{\alpha^*}\right]\right).$$

The limit of strong interactions reached at short distances reads

$$h^{(2)}(0) = \frac{4\left|\frac{\beta\gamma\delta}{\alpha}\right|^2 + |\delta|^4 - 4\left|\frac{\delta}{\alpha}\right|^2\Re\left[\alpha\beta^*\gamma^*\delta\right]}{\left|-\gamma\frac{\beta}{\alpha}+\delta\right|^4}. \tag{74}$$

For illustration, we consider a setup in which the incoming beam is flat on a disk of diameter d and zero elsewhere. This could be realized by placing a mask on top of the TMDC layer, where we restrict our attention to a single hole behind which a detector counts all exiting photons without spatial resolution. Any matrix element is symmetric w.r.t. the interchange $\mathbf{r}_1 \leftrightarrow \mathbf{r}_2$, which faciliates the numerics and leads to $h^{(2)}(\mathbf{r}_1,\mathbf{r}_2) = h^{(2)}(|\mathbf{r}_1-\mathbf{r}_2|)$. The measured quantity would be

$$\bar{g}^{(2)}(\tau=0) = \int d\mathbf{r}_1 \int d\mathbf{r}_2 h^{(2)}(|\mathbf{r}_1-\mathbf{r}_2|) = \int_{\mathbf{r}_1,\mathbf{r}_2\in\text{disk}} d\mathbf{r}d\mathbf{R}h^{(2)}(|\mathbf{r}|)$$
$$= 2\pi\int_0^d dr\ r\cdot 2\left[\left(\frac{d}{2}\right)^2 \arccos\left(\frac{r}{d}\right) - \frac{r}{2}\sqrt{\left(\frac{d}{2}\right)^2 - \left(\frac{r}{2}\right)^2}\right]\cdot h^{(2)}(r) \tag{75}$$

where we moved to center of mass coordinates $\mathbf{r} = \mathbf{r}_2 - \mathbf{r}_1$, $\mathbf{R} = (\mathbf{r}_1+\mathbf{r}_2)/2$ (Jacobi-determinant is 1). In the second line the inner integral depends on the outer and maps out two circle segments (therefore a factor of 2) of height $\left(\frac{d}{2}\right) - r/2$ each, the outer angular integral can then be performed explicitly. The limits of integration are $[0; d]$ because the relative coordinate can be as long as the detector's diameter. It makes sense to normalize to the detector area and define

$$g^{(2)}(\tau=0) = \frac{\bar{g}^{(2)}(\tau=0)}{\pi^2\left(\frac{d}{2}\right)^4} = \frac{64}{\pi d}\int_0^d dr\ r\cdot\left[\left(\frac{d}{2}\right)^2 \arccos\left(\frac{r}{d}\right) - \frac{r}{2}\sqrt{\left(\frac{d}{2}\right)^2 - \left(\frac{r}{2}\right)^2}\right]\cdot g^{(2)}(r) \tag{76}$$

with $g^{(2)}(\tau=0) = 1$ for the noninteracting case. We then define define the distance $R_\text{b}^{(\text{ph})}$ as the spot diameter at which the function $g^{(0)}(0)$ has dropped to $\frac{1}{2}$. The analogous definition applies to $R_\text{b}^{(\text{X})}$, which measures the excitonic rather than the photonic correlation on the basis of $\left\langle \hat{X}_s^\dagger(\mathbf{r}_1)\hat{X}_s^\dagger(\mathbf{r}_2)\hat{X}_s(\mathbf{r}_1)\hat{X}_s(\mathbf{r}_2)\right\rangle$. The results of this calculation are shown in Fig. 3 of the main text.

Supplementary References

[1] Splendiani, A. *et al.* Emerging Photoluminescence in Monolayer MoS_2. *Nano Lett.* **10**, 1271-1275 (2010).

[2] Srivastava, A. & Imamoğlu, A. Signatures of Bloch-Band Geometry on Excitons: Nonhydrogenic Spectra in Transition-Metal Dichalcogenides. *Phys. Rev. Lett.* **115**, 166802 (2015).

[3] Zhou, J., Shan, W.-Y., Yao, W. & Xiao, D. Berry Phase Modification to the Energy Spectrum of Excitons. *Phys. Rev. Lett.* **115**, 166803 (2015).

[4] Cudazzo, P., Tokatly, I. V. & Rubio, A. Dielectric screening in two-dimensional insulators: Implications for excitonic and impurity states in graphane. *Phys. Rev. B* **84**, 085406 (2011).

[5] Andryushin, E., Keldysh, L., Sanina, V. & Silin, A. Electron-hole liquid in thin semiconductor films. *Zh. Eksp. Teor. Fiz.* **79**, 1509–1517 (1980).

[6] Chernikov, A. *et al.* Exciton Binding Energy and Nonhydrogenic Rydberg Series in Monolayer WS_2. *Phys. Rev. Lett.* **113**, 076802 (2014).

[7] Keldysh, L. V. Coulomb interaction in thin semiconductor and semimetal films. *J. Exper. Theor. Phys. Lett.* **29**, 716–719 (1979).

[8] Rytova, N. S. Screened potential of a point charge in a thin film. MSU Physics Bulletin **3**, 30 (1967).

[9] Chaplik, A. V. & Entin, M. V. Charged impurities in very thin layers. *Zh. Eksp. Teor. Fiz.* **61**, 2496–2503 (1971).

[10] Essin, A. M. & Griffiths, D. J. Quantum mechanics of the $1/x^2$ potential. *Am. J. Phys.* **74**, 109–117 (2006).

[11] Pikovski, A. Note on a differentiation formula, with application to the two-dimensional Schrödinger equation. *PloS one* **12**, e0171444 (2017).

[12] Zaslow, B. & Zandler, M. E. Two-Dimensional Analog to the Hydrogen Atom. *Am. J. Phys.* **35**, 1118–1119 (1967).

[13] Xiao, D., Liu, G.-B., Feng, W., Xu, X. & Yao, W. Coupled Spin and Valley Physics in Monolayers of MoS_2 and Other Group-VI Dichalcogenides. *Phys. Rev. Lett.* **108**, 196802 (2012).

[14] Carusotto, I. & Ciuti, C. Quantum fluids of light. *Rev. Mod. Phys.* **85**, 299–366 (2013).

[15] Gaul, C. *et al.* Resonant Rydberg Dressing of Alkaline-Earth Atoms via Electromagnetically Induced Transparency. *Phys. Rev. Lett.* **116**, 243001 (2016).

[16] Wang, G. *et al.* Giant Enhancement of the Optical Second-Harmonic Emission of WSe_2 Monolayers by Laser Excitation at Exciton Resonances. *Phys. Rev. Lett.* **114**, 097403 (2015).

[17] Fleischhauer, M. & Lukin, M. Dark-State Polaritons in Electromagnetically Induced Transparency. *Phys. Rev. Lett.* **84**, 5094 (2000).

[18] Le Roy, R. Long-Range Potential Coefficients From RKR Turning Points: C_6 and C_8 for $B(3\Pi^+_{Ou})$-State Cl_2, Br_2, and I_2. *Can. J. Phys.* **52**, 246–256 (1974).

[19] Rice, P. R. & Carmichael, H. J. Single-atom cavity-enhanced absorption. I. Photon statistics in the bad-cavity limit. *IEEE J. Quantum Electron.* **24** 1351–1366 (1988).
